# Transcriptomic Analysis and Machine Learning Identify Cross-Pathogen Biomarkers for Bacterial and Parasitic Infections in Silver Pomfret (*Pampus argenteus*)

**DOI:** 10.3390/ani16101510

**Published:** 2026-05-14

**Authors:** Yunkang Wu, Yuanbo Li, Ting Chen, Wuqiang Xia, Yajun Wang, Xiaojun Yan, Jiabao Hu

**Affiliations:** 1 School of Marine Sciences, Ningbo University, Ningbo 315211, China; 2 Key Laboratory of Applied Marine Biotechnology, Ningbo University, Ministry of Education, Ningbo 315211, China; 3 Key Laboratory of Marine Biotechnology of Zhejiang Province, Ningbo University, Ningbo 315211, China; 4 Xiangshan Aquatic Seed Industry Innovation Research Institute, Ningbo 315211, China

**Keywords:** *Pampus argenteus*, *Cryptocaryon irritans*, *Nocardia seriolae*, *Photobacterium damselae* subsp. *damselae*, cross-pathogen biomarker

## Abstract

Silver Pomfret is increasingly threatened by many diseases under intensive artificial culturing conditions, yet conserved host biomarkers across different infections remain poorly defined. In this study, we integrated transcriptomic datasets from independent infections with *Cryptocaryon irritans*, *Nocardia seriolae*, and *Photobacterium damselae* subsp. *damselae* to identify shared host-response genes. By combining differential expression analysis with weighted gene co-expression network analysis, we prioritized six candidate genes associated with cross-pathogen infection responses. Random Forest and support vector machine analysis further supported their classification potential across the three infection models. Phylogenetic and structural analyses provided additional evidence for the conserved annotation of these proteins. GSVA-based signature analysis supported the cross-pathogen discriminatory capacity of the six-gene panel and suggested context-dependent contributions of individual genes across infection models. Immune signature analysis indicated distinct host immune response patterns under different pathogenic challenges, and candidate genes showed positive associations with inferred T cell-related signatures. Upstream regulatory prediction identified CTCF and the miR-17/20/93 family as potential regulators of these genes. Quantitative real-time PCR of the kidney further highlighted *canx*, *rnd3*, and *angptl4* as the most robust infection-responsive candidates, with consistent temporal expression patterns observed from 0 to 24 h post-infection. These findings suggest a potential cross-pathogen host-response pattern in Silver Pomfret and provide preliminary support for future exploration of molecular markers for disease monitoring in aquaculture.

## Simple Summary

Silver Pomfret in aquaculture is frequently affected by infections caused by the Gram-negative bacterium *Photobacterium damselae* subsp. *damselae*, the Gram-positive bacterium *Nocardia seriolae*, and the protozoan parasite *Cryptocaryon irritans*, but cross-pathogen molecular markers remain poorly defined. By comparing transcriptomic responses and immune infiltration analysis across these three infection conditions, we identified six shared responsive genes, among which *canx*, *angptl4*, and *rnd3* showed relatively consistent changes during infection and discriminated infected from healthy fish. These genes represent candidate cross-pathogen response biomarkers and have potential for the future aquaculture industry of Silver Pomfret.

## 1. Introduction

Silver Pomfret is recognized as a premium marine fish species with high nutritional value and significant market demand in East Asian aquaculture [1]. However, its large-scale artificial culture remains challenging, especially due to frequent disease outbreaks [1,2,3]. Presently, major pathogens include *Nocardia seriolae* (*N. seriolae*), *Photobacterium damselae* subsp. *damselae* (*P. damselae*) and the parasite *Cryptocaryon irritans* (*C. irritans*) [1,2,4], all of which tend to cause outbreaks during summer months [4,5,6]. *N. seriolae* causes chronic granulomatous disease characterized by numerous white nodules in the liver and kidney, which severely compromises the internal organ functions over a long-term period [1]. *P. damselae* induces acute hemorrhagic septicemia with rapid tissue necrosis and leads to total stock loss within a few days of infection [7]. *C. irritans* targets the skin and gills to cause white spot disease which disrupts the mucosal barrier and leads to respiratory failure in the host [2]. Therefore, identifying core host-response biomarkers is essential for overcoming these production bottlenecks.

Previous molecular investigations into Silver Pomfret have primarily focused on host responses to individual pathogens. Most transcriptomic studies characterize the regulation of immune genes during a single pathogen infection [1,2,4]. However, research on the cross-pathogen and broad-spectrum regulatory mechanisms remain entirely blank. This knowledge gap prevents the identification of host biomarkers that can monitor host health across multiple types of pathogenic stress. There is an urgent need to identify core biomarkers that respond to various pathogens, which is helpful for understanding the fundamental immune architecture and useful for health monitoring under the complex farming conditions for Silver Pomfret in complex aquaculture systems.

In this study, we performed an integrative comparative transcriptomic analysis using datasets from independent infections with *C. irritans*, *N. seriolae*, and *P. damselae*. Differential expression analysis and weighted gene co-expression network analysis (WGCNA) were combined to identify shared candidate genes associated with infection responses. These genes were further prioritized using machine learning approaches, including Random Forest (RF) and support vector machine (SVM). We further applied GSVA-based signature analysis and perturbation analysis to evaluate the collective infection-response pattern of the identified gene panel and to assess the relative contribution of individual genes within this signature. Also, we conducted phylogenetic and structural analyses to support gene annotation, predicted potential upstream regulators, and evaluated their associations with inferred immune signatures. Finally, RT-qPCR was used to validate the expression patterns of key candidate genes. This study provides a comparative framework for identifying conserved host-response biomarkers and offers candidate molecular markers for host-based infection monitoring in Silver Pomfret aquaculture.

## 2. Materials and Methods

### 2.1. Transcriptome Analysis to Identify Cross-Pathogen Biomarkers

Transcriptomic datasets of Silver Pomfret were retrieved from the NCBI, including *C. irritans* infection (PRJNA884829), *N. seriolae* infection (PRJNA948898), *P. damselae* infection (PRJNA975702), and low-temperature stress (PRJNA783750). These datasets were generated in previous studies, in which sequencing depth and primary processing procedures were reported [2,3,8,9]. In this study, raw count matrices were used for differential expression analysis, whereas TPM values were used only for expression quantification and visualization. Functional annotation was performed by protein-sequence-based homology mapping against zebrafish (*Danio rerio*) reference proteins using DIAMOND, yielding 3662, 7812, 9540, and 11,062 unique annotated orthologs for the *C. irritans*, *N. seriolae*, *P. damselae*, and low-temperature datasets, respectively. Differential expression analysis was conducted using DESeq2 in R, and genes with |log_2_ fold change| > 1 and adjusted *p* < 0.05 were defined as differentially expressed genes. The details of samples are shown in Table S1. Functional enrichment analyses, including Gene Ontology (GO) and Kyoto Encyclopedia of Genes and Genomes (KEGG), were performed using the “clusterProfiler” package. Weighted gene co-expression network analysis (WGCNA) was independently performed for each transcriptome dataset. Module eigengenes (MEs) were used to calculate Pearson correlation coefficients with infection status. Genes from significant modules were intersected with shared DEGs and visualized using Venn diagrams. Gene set enrichment analysis (GSEA) was further performed to assess biological pathways associated with infection-responsive gene sets. All statistical analyses and visualizations were performed using R version 4.4.3.

### 2.2. Machine Learning Evaluation of Candidate Biomarker Features

To evaluate the classification performance of the candidate biomarkers, machine learning analyses were performed in R using the “randomForest,” “e1071,” and “glmnet” packages. Random Forest (RF) and support vector machine (SVM) models were trained on the infection datasets. Model hyperparameters were optimized by five-fold cross-validation within the training data. Cross-pathogen generalizability was assessed using leave-one-pathogen-out (LOPO) validation, in which two infection datasets were used for training and the remaining dataset was used for testing, generating three train–test combinations. Model performance was summarized by ROC curves and AUC values, with 95% confidence intervals estimated from 1000 bootstrap resamples. DeLong’s test was used to compare AUCs between competing models on the same test set. To reduce overfitting, model complexity was controlled during training. Permutation importance in the RF framework was used to quantify the contribution of individual genes, and LASSO logistic regression was applied as an independent feature-selection approach to evaluate the stability of the selected biomarker panels.

The discriminatory performance of the candidate biomarkers was further evaluated using receiver operating characteristic (ROC) curves generated with the “pROC” package. The area under the curve (AUC) was calculated for each gene. In addition, a multi-gene classification model was established using logistic regression analysis. The performance of this integrated model was compared with that of individual gene models by ROC analysis to identify the optimal gene combination for Silver Pomfret health monitoring.

### 2.3. Identification and Characterization of Candidate Genes

Protein sequences from *Mus musculus*, *Homo sapiens*, *Larimichthys crocea*, *Danio rerio*, *Thunnus albacares*, *Thunnus thynnus*, and *Thunnus maccoyii* were retrieved from the NCBI database. The Silver Pomfret gene sequences were obtained from transcriptome-derived sequences. ORF regions were predicted using the ORF Finder in TBtools-II [10] and translated into protein sequences. Gene identity was validated by BLAST and BLASTP in NCBI searches against the NCBI database, and further confirmed through phylogenetic analysis and conserved domain conservation. Sequence alignment and phylogenetic analysis were conducted using MEGA 12 software. The phylogenetic tree was constructed using the Maximum Likelihood (ML) algorithm based on the Jones–Taylor–Thornton (JTT) model. A bootstrap test with 1000 replicates was performed to assess the reliability of the tree branches. Conserved domains of the target proteins were analyzed using the Batch CD-Search Tool on NCBI with the following settings: data source = CDD, E-value cut-off = 0.01, composition-corrected scoring = applied, low-complexity region filtering = not filtered, and maximum number of alignments = 500. The visualization of the phylogenetic tree and gene structures was completed using the Chiplot [11] online tool (https://www.chiplot.online, accessed on 11 May 2026).

Homology modeling for the three-dimensional structures of the candidate proteins was performed using Swiss-model and visualized with PyMOL 3.0.3. Secondary structure prediction was conducted through the SOPMA web server. Meanwhile the physicochemical properties of the candidate genes were predicted using the ProtParam tool on the ExPASy server. Subcellular localization was determined using the WoLF PSORT. Transmembrane domains and signal peptides were predicted using the TMHMM 2.0 and SignalP 6.0 servers respectively.

### 2.4. GSVA Scoring and In Silico Perturbation Analysis of the Six-Gene Signature

To assess the discriminatory capacity of the six-gene signature at the sample level, gene set variation analysis (GSVA) was performed in each cohort using a custom gene set comprising *cbx7*, *vdr*, *fbxo2*, *rnd3*, *canx*, and *angptl4*. A GSVA score was calculated for each sample from the normalized expression matrix and compared between infected and control groups in the *C. irritans*, *N. seriolae*, and *P. damselae* cohorts. Diagnostic performance was then evaluated by ROC analysis, and the AUC was used to summarize classification accuracy.

We performed an in silico perturbation analysis to estimate the contribution of each gene to the six-gene signature. In each cohort, one gene was perturbed at a time while the remaining five genes were left unchanged. The expression values of the target gene were replaced using four alternative strategies, including the control-group median, the control-group mean, a random draw from control samples, and a conditional Gaussian estimate derived from the control data, to approximately preserve the covariance structure among genes. GSVA scores and ROC curves were then recalculated from each perturbed matrix. The consistency of gene contribution rankings across the four perturbation strategies was compared to evaluate robustness, and 500 bootstrap repetitions were performed for the stochastic procedures.

The effect of perturbation on classification performance was quantified as

$$\Delta {AUC}_{g}={AUC}_{\mathrm{perturbed},g}-{AUC}_{baseline}$$

${AUC}_{baseline}$ denotes the AUC of the original gene’s signature and ${AUC}_{perturbed,g}$ represents the AUC after the perturbation of gene. More negative $\Delta {AUC}_{g}$ values indicate a greater reduction in diagnostic performance after perturbation.

To quantify the effect of perturbation on infected–control separation, the baseline signature difference was defined as

$$D_{baseline}=\left|mean\left(S_{infected}\right)-mean\left(S_{control}\right)\right|$$

and the perturbed difference for gene g was defined as

$$D_{perturbed,g}=|mean(S_{infected,g})-mean(S_{control,g})|$$

The rollback percentage was calculated as

$$Rollback,\;g\;(\%)=[(D_{baseline}-D_{perturbed,g})/D_{baseline}]\times 100$$

Higher positive rollback values indicate a greater reduction in the original infected–control separation after perturbation, whereas negative values indicate that the separation increased rather than decreased. Gene contribution was interpreted by jointly considering ΔAUC, rollback percentage, and the consistency of these effects across cohorts.

### 2.5. Construction of Transcription Factor–miRNA and Protein Interaction Networks

To investigate the upstream regulatory landscape of the candidate genes, we predicted potential transcription factors using the JASPAR and ChIP-Atlas databases. The intersection of these transcription factors from both databases was visualized through Venn diagrams and Upset plots with R. Targeted miRNA sequences and their respective binding sites for the candidate genes were identified using the TargetScanFish 6.2 platform. The regulatory interactions between transcription factors and miRNAs were visualized using Cytoscape 3.10.4. Finally, the PPI network for the candidate genes was established based on the topological connectivity patterns identified in the WGCNA from the three infection datasets.

### 2.6. Immune Cell Infiltration Analysis

The relative abundance of 16 immune cell types was estimated based on established molecular markers in zebrafish [12]. The single sample gene set enrichment analysis (ssGSEA) was employed to calculate the immune infiltration scores for both control and infected groups with the “GSVA” package. Significant differences in immune cell populations before and after infection were determined using *t* tests. Furthermore, Pearson correlation analysis was conducted to evaluate the internal associations among the 16 distinct immune cell types across the three infection models. Finally, the Mantel test was applied to analyze the correlations between the identified candidate genes and the varying immune cell populations to identify potential gene cell interactions. The correlations between candidate genes and immune cells were visualized in Chiplot (Table S2) [13].

### 2.7. Real-Time Quantitative PCR (RT-qPCR) Validation

The cDNA samples were acquired from our previous studies [1,3]. RT-qPCR was conducted on an Eppendorf thermal cycler (Stevenage, UK). All specific primer sequences designed with Primer Premier 6 for these amplification reactions are detailed in Table 1. The beta-actin demonstrated the optimal amplification efficiency and expression stability among three evaluated internal controls. This assessment was performed using Normfinder software version 0.953 to ensure reliable data normalization. The amplification was performed according to the Takara Kit (Takara, Tokyo, Japan). Relative expression levels were calculated using the 2^−ΔΔCt^ method and were analyzed by one-way analysis of variance (ANOVA).

## 3. Results

### 3.1. Comparative Differential Expression and Functional Enrichment Analyses Across Three Pathogen Infections

**Figure 1:**
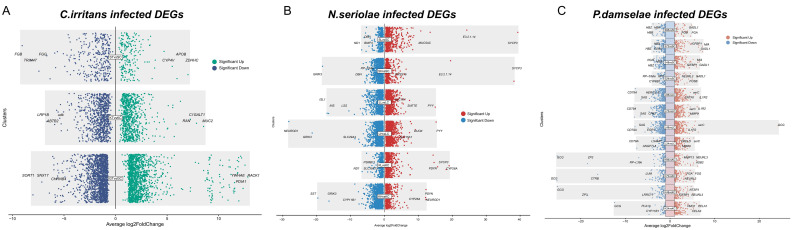
Identification of DEGs across three pathogenic infection transcriptomes. The criteria for identifying significant genes were established as |log_2_ fold change| > 1 and adjusted *p* < 0.05.

**Figure 2:**
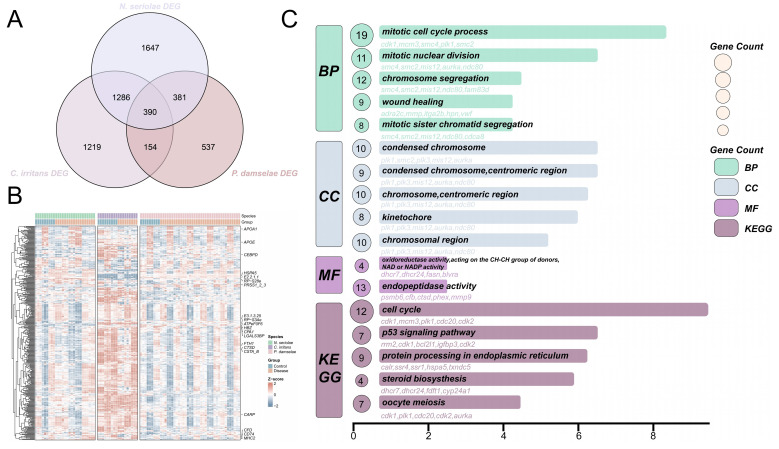
Intersection and functional enrichment analysis of DEGs. (**A**) Venn diagram demonstrating the intersection of DEGs among the three pathogenic infection transcriptomes. (**B**) Hierarchical clustering heatmap of the 390 core shared DEGs. The top 20 most significant genes are labeled on the right. Colors represent relative expression levels standardized by Z-score in control and infected groups. (**C**) GO and KEGG functional enrichment results of the core shared DEGs. Different colors designate the distinct classifications including BP, CC, MF, and specific KEGG pathways. Colors indicate BP, CC, MF, and KEGG categories. Numbers in the circles represent gene counts for each enriched term, and bar length indicates enrichment significance. The top 5 enriched genes for each pathway are listed below.

Based on transcriptomic analysis (Figure 1), the intersection of these independent datasets yielded a conserved core of 390 DEGs that were consistently altered regardless of the pathogen type (Figure 2A). Hierarchical clustering of this core gene set demonstrated a high degree of expression synchrony. Representative genes involved in cellular stress and immune recognition showed a highly consistent expression pattern (Figure 2B).

Based on function analysis (Figure 2C), the GO result showed that these shared DEGs mainly enriched replication and repair mechanisms, mitotic cell cycle process, condensed chromosome and so on. KEGG results showed that the core DEGs significantly enriched several critical pathways including the cell cycle (dre04110) and p53 signaling pathways (dre04115).

### 3.2. Weighted Gene Co-Expression Network Analysis and Identification of Infection-Associated Modules

**Figure 3:**
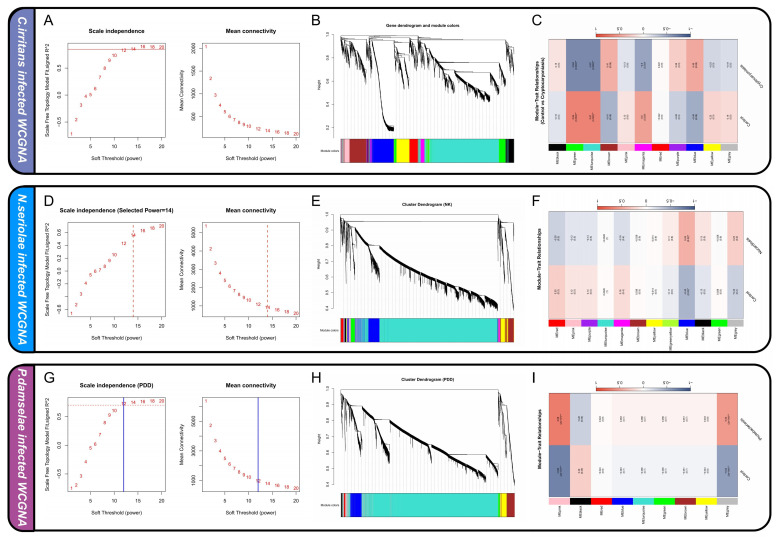
WGCNA across three pathogenic infection models. (**A**,**D**,**G**) Scale independence and mean connectivity evaluation for optimal soft-thresholding power selection in *C. irritans*, *N. seriolae*, and *P. damselae* infections, respectively. (**B**,**E**,**H**) Hierarchical clustering dendrograms of genes with corresponding module colors assigned below. (**C**,**F**,**I**) Module–trait relationship heatmaps illustrating the correlations between identified gene modules and clinical infection status. Color reflects the strength of the correlation, with darker shades representing stronger associations. The “*” indicates the statistical significance level of the correlation, where the number of “*” denotes stronger statistical significance (three level significance: “*” < “**” < “***”).

Based on the WGCNA results, for *C. irritans*, a power of 14 was selected to ensure the network reached a scale-free topology model fit (R^2^) of approximately 0.85. Similarly, power values of 14 and 12 were applied to the *N. seriolae* and *P. damselae* transcriptomes respectively to facilitate the construction of robust hierarchical clustering trees (Figure 3A,D,G). Based on the topological overlap measure, genes with similar expression patterns were grouped into distinct co-expression modules, which are represented by different colors in the dendrograms (Figure 3B,E,H). The correlation between these co-expression modules and the infection status was evaluated to pinpoint the gene sets most relevant to the disease progression. In the *C. irritans* infection group, the green module, the turquoise module and the magenta module exhibited the highest positive correlations with the diseased state (Figure 3C). For the host response to *N. seriolae* (Figure 3F), the blue module demonstrated a significant association with the occurrence of nocardiosis. In the analysis of *P. damselae* infection (Figure 3I), the pink module showed a strong positive correlation with the diseased phenotype, while the gray module also displayed a significant relationship. These identified modules were considered as the candidate gene pools for the subsequent analysis.

### 3.3. Identification and Machine Learning-Based Evaluation of Universal Biomarker Candidates

**Figure 4:**
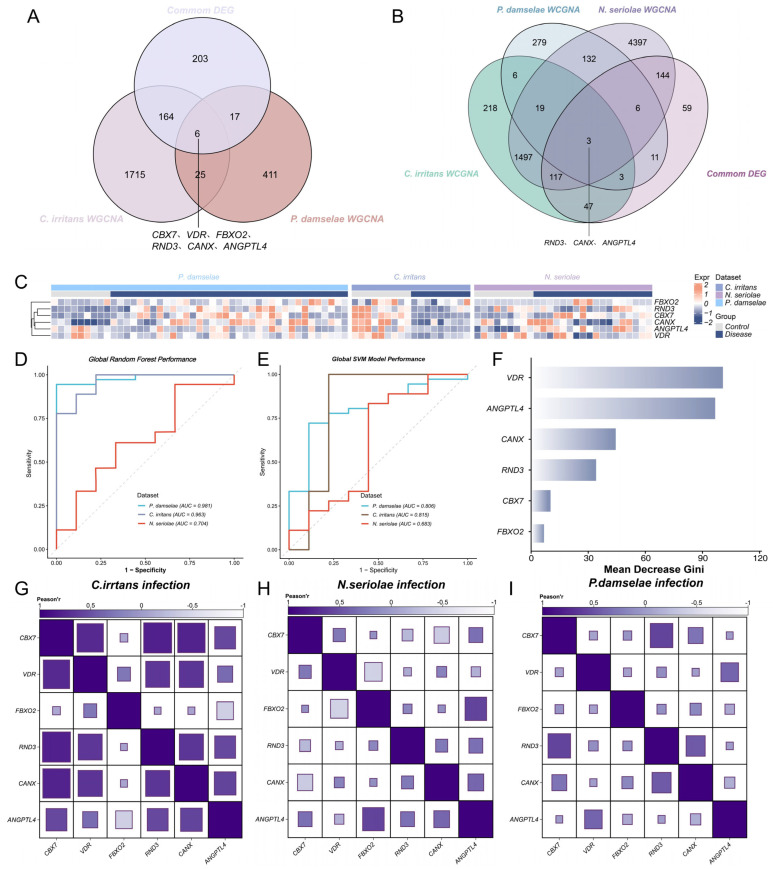
Identification and machine learning evaluation of candidate hub genes. (**A**) Venn diagram illustrating the intersection of the core shared DEGs with the significant WGCNA modules from *C. irritans* and *P. damselae* infections. (**B**) Venn diagram demonstrating a stricter intersection incorporating the WGCNA module from the *Nocardia seriolae* infection. (**C**) Expression heatmap of the 6 candidate hub genes across the three different infection transcriptomes. (**D**,**E**) ROC evaluating the diagnostic accuracy of the global RF and SVM models, respectively. (**F**) Feature importance ranking of the candidate genes based on the RF algorithm. (**G**–**I**) Correlation analysis of the six hub genes under different pathogen infections. The gray dashed diagonal line represents the line of no discrimination, indicating the performance of a random classifier (AUC = 0.5).

Based on WGCNA analyses, we obtained a refined panel of six critical candidate genes: *cbx7*, *vdr*, *fbxo2*, *rnd3*, *canx*, and *angptl4* (Figure 4A). Notably, the direct intersection among all three diseases and the core common DEGs further identified *rnd3*, *canx*, and *angptl4* as the most universal targets shared across all infection conditions (Figure 4B). The expression patterns of these six candidate genes across the three disease models are shown in the heatmap (Figure 4C). All six genes were markedly downregulated in the *C. irritans* model, whereas they showed an overall upregulated tendency in the *P. damselae* and *N. seriolae* infection models.

Receiver operating characteristic (ROC) analysis demonstrated that the RF model outperformed the SVM model in discriminating infected from control samples. The global RF classifier achieved excellent predictive performance, with area under the curve (AUC) values of 0.963 and 0.981 in the two training datasets. Importantly, the model retained acceptable predictive ability in the independent *N. seriolae* validation set, with an AUC of 0.704 (Figure 4D,E). The ROC of individual genes in the three infection groups were shown in Figure S1.

The six-gene model maintained consistent discriminatory performance in each round, with AUC values of 0.891, 0.827, and 0.722 when *P. damselae*, *C. irritans*, and *N. seriolae* were used as the respective test sets (Figure S2A). Consistent with the RF results, a LASSO-logistic regression model also achieved high classification performance (AUC = 0.864), and no significant difference was detected between the two classifiers by DeLong’s test (*p* = 0.598) (Figure S2B). By contrast, evaluation in an external low-temperature stress dataset yielded only limited discrimination (AUC = 0.674).

Model stability was further supported by repeated resampling analysis, in which the observed LOPO AUC remained significantly higher than the empirical null distribution generated from 1000 repetitions (*p* < 0.001; Figure S2D). Within the RF framework, feature importance ranking based on mean decrease in Gini identified *vdr* and *angptl4* as the most influential variables contributing to classification accuracy (Figure 4F). This ranking was corroborated by cross-validated permutation importance analysis (Figure S2E). Individual ROC analyses likewise showed that most hub genes displayed strong discriminatory ability in the training cohorts and retained measurable predictive value in the independent validation dataset.

Based on Pearson correlation analyses, in the *C. irritans* group, *canx* and *angptl4* were positively correlated with nearly all other hub genes except *fbxo2*, while *fbxo2* showed an obvious negative correlation with *angptl4* (Figure 4G); in the *N. seriolae* group, *fbxo2* and *angptl4* were positively correlated (Figure 4H), *angptl4* showed a positive correlation with *vdr*, and *rnd3* exhibited a relatively strong positive correlation with *cbx7* (Figure 4I).

### 3.4. Gene Set Enrichment Analysis of the Core Genes Under Diverse Infections

**Figure 5:**
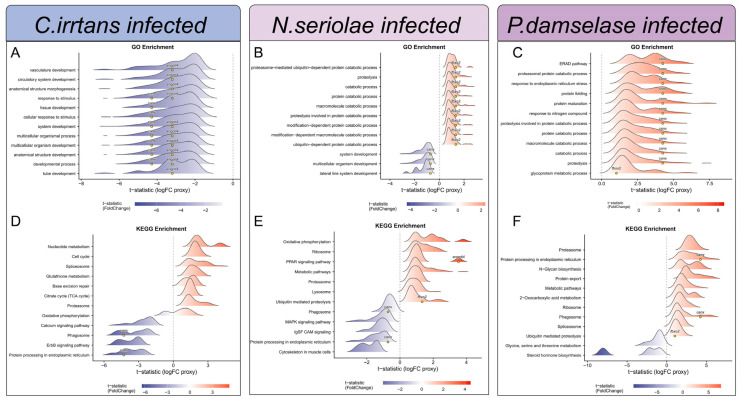
Gene set enrichment analysis of the identified hub genes across three pathogenic challenges. (**A**–**C**) Ridgeline plots of the GSEA results for GO terms in three infection groups. (**D**–**F**) Ridgeline plots of the GSEA results for KEGG pathways.

Based on the GSEA results, ridge plot analysis revealed distinct patterns of pathway activation and suppression across the three transcriptomes (Figure 5). In *C. irritans* infection, GO terms showed an overall downregulated trend in fundamental biological processes, including vasculature development (GO:0001944) and response to stimulus (GO:0050896). KEGG analysis showed a mixed pattern, with cell cycle (dre04110) enriched among upregulated pathways, whereas phagosome (dre04145) and protein processing in endoplasmic reticulum (dre04141) were significantly downregulated. Meanwhile in *N. seriolae* infection, enrichment analysis indicated a shift toward cellular degradation and protein turnover. GO terms were mainly enriched in proteolysis (GO:0006508) and proteasome-mediated ubiquitin-dependent protein catabolic process (GO:0043161), whereas broader developmental processes such as system development (GO:0048731) were suppressed. KEGG results were consistent with this pattern, showing upregulation of proteasome (dre03050) and lysosome (dre04142), alongside downregulation of phagosome (dre04145) and protein processing in endoplasmic reticulum (dre04141). By contrast, *P. damselae* infection was characterized by broad activation of stress-response and protein homeostasis pathways. GO enrichment highlighted response to endoplasmic reticulum stress (GO:0034976) and protein folding (GO:0006457). KEGG analysis further supported this pattern, with strong enrichment of protein processing in endoplasmic reticulum (dre04141) and proteasome (dre03050), while steroid hormone biosynthesis (dre00140) and glycine, serine and threonine metabolism (dre00260) were downregulated. Enrichment results for the six hub genes are provided in Table S3 and Figure S3.

### 3.5. Identification and Characterization of Hub Genes

Phylogenetic analysis showed that Silver Pomfret Angptl4, Fbxo2, Vdr, Cbx7, Canx, and Rnd3 clustered with their respective orthologs from other vertebrates. And these proteins retained the characteristic domains reported for each gene family (Figure S4). The basic features of the six hub proteins are summarized in Table S4. And secondary structure prediction and homology modeling also supported the overall structural integrity of these proteins (Table S5).

### 3.6. GSVA-Based Evaluation and In Silico Perturbation Analysis of the Six-Gene Signature

**Figure 6:**
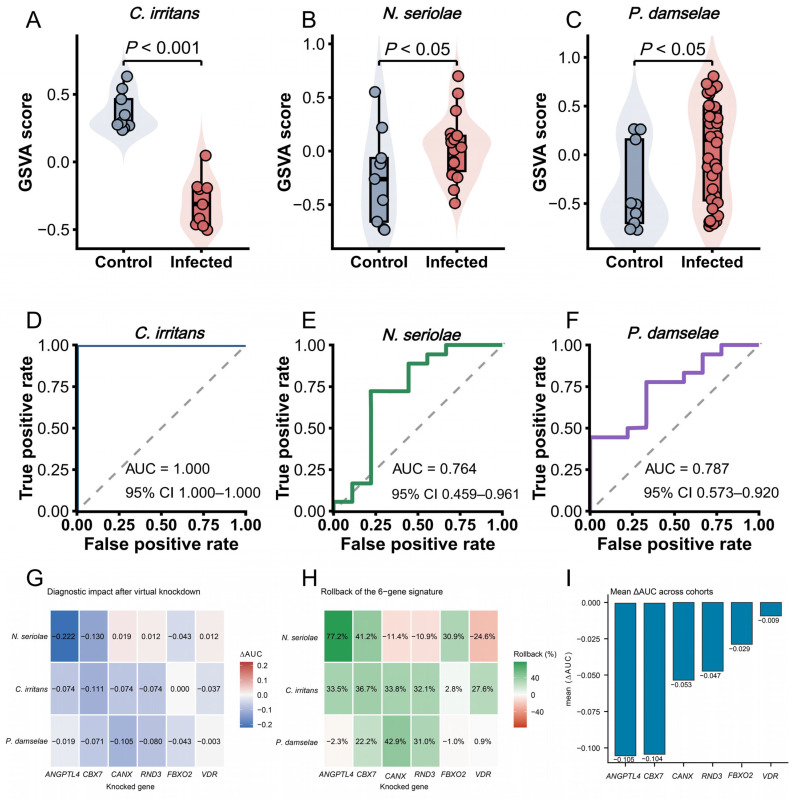
GSVA-based evaluation and in silico perturbation analysis of the six-gene signature. (**A**–**C**) GSVA score distributions in control and infected samples from the *C. irritans*, *N. seriolae*, and *P. damselae* cohorts. (**D**–**F**) ROC curves of the baseline six-gene GSVA signature in the three cohorts, with the AUC and 95% confidence interval (CI) shown in each panel. (**G**) Heatmap of ΔAUC after single-gene perturbation. (**H**) Heatmap of rollback percentage after perturbation. (**I**) Mean ΔAUC of each gene across cohorts. The gray dashed diagonal line represents the line of no discrimination, indicating the performance of a random classifier (AUC = 0.5).

GSVA showed that the six-gene signature significantly separated infected and control samples in the *C. irritans* cohort (*p* < 0.001), the *N. seriolae* cohort (*p* < 0.05), and the *P. damselae* cohort (*p* < 0.05). ROC analysis of the baseline GSVA score yielded an AUC of 1.000 in *C. irritans*, 0.764 in *N. seriolae*, and 0.787 in *P. damselae* (Figure 6).

In the *P. damselae* cohort, perturbation of *canx* produced the largest rollback of the six-gene signature (42.9%), followed by *rnd3* (31.0%) and *cbx7* (22.2%). Perturbation of *vdr* had little effect (0.9%), whereas angptl4 and fbxo2 showed negative rollback values. Meanwhile in the *C. irritans* cohort, *cbx7* showed the highest rollback (36.7%). Perturbation of *angptl4*, *canx*, and *rnd3* produced similar rollback values (33.5%, 33.8%, and 32.1%, respectively), whereas *fbxo2* showed the smallest effect (2.8%). For the *N. seriolae* cohort, *angptl4* showed the strongest effect, with a rollback of 77.2%, followed by *cbx7* (41.2%) and *fbxo2* (30.9%). In contrast, perturbation of *canx*, *rnd3*, and *vdr* resulted in negative rollback values. Across the three cohorts, the perturbation effects differed among genes and infection models (Figure 6G,H and Table S6). The largest overall decreases in signature performance were observed for *angptl4* and *cbx7*, whereas *vdr* showed the smallest overall effect (Figure 6I). Four alternative strategies (median, mean, random control draw, and conditional Gaussian) were further compared across the three cohorts. Overall, cross-strategy concordance was highest in *N. seriolae*, remained generally high in *P. damselae*, and was lower in *C. irritans*. Consistently, the correlation heatmap and rollback-based rank tiles showed relatively stable gene ordering across strategies in *N. seriolae* and *P. damselae*, whereas *C. irritans* displayed more evident rank shifts (Figure S5A,B). Detailed 500 bootstrap estimates of ΔAUC and rollback for each gene under each perturbation strategy are provided in Table S7.

### 3.7. Construction of Upstream Regulatory Networks and Topological Interaction Analysis

**Figure 7:**
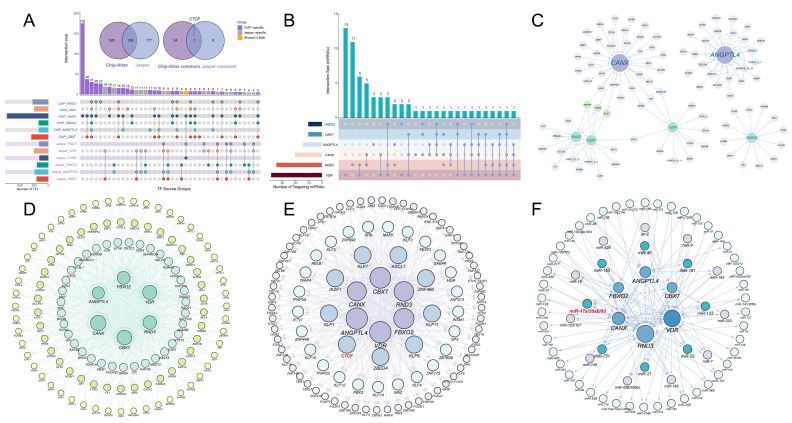
Construction of upstream regulatory and protein interaction networks for the candidate genes. (**A**) UpSet plot of the predicted transcription factors from the JASPAR and ChIP-Atlas databases. The right inset Venn diagram isolates the shared transcription factors regulating all six candidate genes in both databases with CTCF explicitly labeled as the sole unique overlapping target. (**B**) UpSet plot of predicted miRNAs targeting the 6 candidate genes. (**C**) Protein interaction network constructed based on the topological connectivity derived from the three WGCNA transcriptomes. (**D**,**E**) Visualizations of the transcription factor regulatory networks. (**F**) Visualization of the comprehensive miRNA regulatory network. In the UpSet plot, different colors denote different candidate genes. In the Venn plot, purple and blue indicate the ChIP-Atlas and JASPAR databases, respectively. Arrows in the network represent the interactions among the indicated factors.

ChIP-Atlas predicted 35 common transcription factors targeting the full set of core genes, whereas JASPAR identified nine common regulators (Figure 7A). Cross-comparison of these two datasets yielded a single shared transcription factor, CTCF. Regulatory networks constructed from both databases consistently highlighted CTCF as a central upstream node connected to multiple candidate genes (Figure 7D,E).

The post-transcriptional regulatory landscape was further assessed by predicting miRNAs targeting the six core genes (Figure 7B), with binding sites summarized in Supplementary Table S8. The UpSet plot illustrated the intersection and distribution of shared and unique miRNAs across the six targets. Network analysis revealed a complex miRNA–gene regulatory architecture, within which the miR-17a/20ab/93 cluster was particularly notable, as it was predicted to target all six core genes simultaneously (Figure 7F).

In Figure 7C, the results indicated distinct connectivity patterns among the candidate genes. *angptl4* and *fbxo2* each formed relatively independent co-expression modules, interacting with unique adjacent genes without shared nodes. In contrast, *canx*, *rnd3*, *cbx7*, and *vdr* formed an interconnected subnetwork with multiple shared co-expressed neighbors. In particular, Canx, Rnd3, and Cbx7 were linked through shared interacting proteins including PHLPP, Tnks, and Plk1, while Canx and Vdr were additionally connected through Kdm4, Tut, and B4galt6.

### 3.8. Immune Infiltration and Correlation Network Analysis

**Figure 8:**
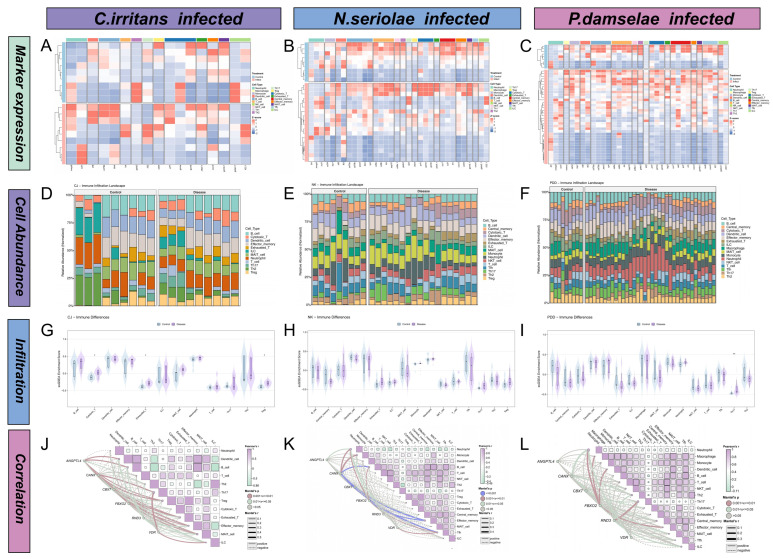
Immune cell infiltration profiles and correlation networks across three infection models. (**A**–**C**) Heatmaps of the expression patterns of specific immune marker genes across the three infection models. (**D**–**F**) Relative immune cell abundance landscapes characterizing the microenvironments. (**G**–**I**) Violin plots detailing the ssGSEA immune infiltration scores for all identified immune cell types. Statistical divergences between comparative groups were assessed utilizing independent *t* tests. (**J**–**L**) Correlation networks integrating Pearson correlation coefficients among distinct immune cells and Mantel test results between the defined candidate genes and specific immune traits. Varying colors represent exact statistical significance boundaries where blue lines indicate *p* < 0.001, purple lines indicate 0.001 < *p* ≤ 0.01, green lines denote *p* < 0.05, and gray lines identify interactions with *p* > 0.05. Specific line formats describe the correlation direction with solid lines establishing positive relationships and dashed lines illuminating negative interactions. Thicker connecting lines directly represent a functionally stronger overall correlation magnitude. Asterisks indicate the level of statistical significance, where * denotes *p* < 0.05 and ** denotes *p* < 0.01.

A total of 17, 30, and 34 immune marker genes were identified in the *C. irritans*, *N. seriolae*, and *P. damselae* models, respectively (Figure 8A–C). Based on these marker genes, the relative abundance of immune cell-related signatures was further estimated (Figure 8D–F). In the *C. irritans* model, Treg-like, Th2, and dendritic cell signatures showed the most obvious changes after infection. In the *N. seriolae* model, pronounced shifts were mainly observed in Th2 and dendritic cell signatures. In the *P. damselae* model, B cell, effector memory, Treg-like, and neutrophil signatures exhibited relatively greater changes.

In Figure 8G–I, Treg-like, Th17-like, T cell, and exhausted T cell signatures were significantly enriched in the *C. irritans* infection model. Similarly, in the *N. seriolae* model, cytotoxic T cell, exhausted T cell, Th17-like, and Treg-like signatures were significantly increased. In contrast, the *P. damselae* model showed a different trend, in which Treg-like, Th17-like, T cell, and exhausted T cell signatures were significantly decreased.

In Figure 8J–L, among the three models, the *N. seriolae* infection showed the strongest overall gene–immune associations, where Angptl4, Canx, and Cbx7 were strongly correlated with neutrophil, monocyte, and dendritic cell signatures. In the *C. irritans* model, Angptl4 and CanX were mainly associated with neutrophil and Th2 signatures. Meanwhile in the *P. damselae* model, Fbxo2 and Rnd3 showed relatively strong positive correlations with myeloid-related signatures.

### 3.9. Expression Validation of Core Genes Across Different Infection Models

**Figure 9:**
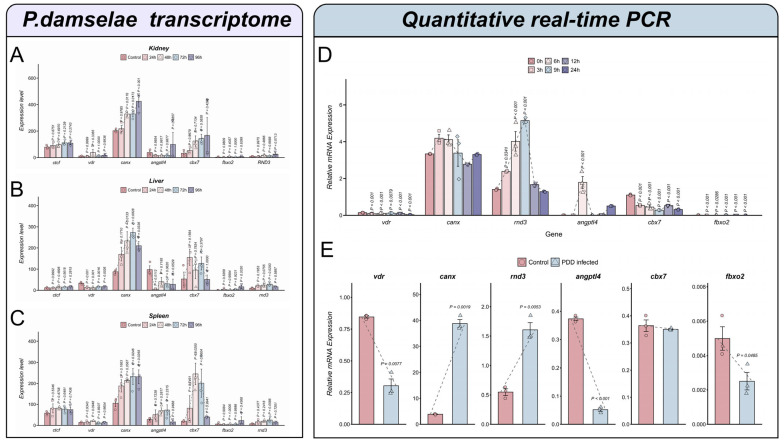
Temporal expression dynamics and quantitative experimental validation of the identified core genes. (**A**–**C**) Temporal transcriptomic expression profiles of the candidate genes and the core transcription factor CTCF in kidney, liver, and spleen tissues during *P. damselae* infection. Distinct bar colors correspond to the different evaluated treatment time points post-infection. (**D**) The expression levels of the candidate genes across various temporal stages of *N. seriolae* infection (*p* < 0.05). (**E**) RT-qPCR of the selected candidate genes under *P. damselae* infection. *p* < 0.05 indicates significant differences and a *p* < 0.01 represents highly significant differences. The dashed line represents the changing trend across treatments.

We analyzed the core genes during *P. damselae* infection across multiple time points (Figure 9A–C). In the immune tissues, the core genes along with the transcription factor CTCF exhibited distinct and significant expression shifts. Specifically, *canx* demonstrated consistent time-dependent upregulation across all three tissues reaching peak expression levels post-infection. The transcription factor CTCF maintained a stable but observable presence across the infection stages. Transcriptomic tissue-level analyses further showed broad downregulation of *cbx7*, *vdr*, *rnd3*, *canx*, and *angptl4* across the gill, kidney, and spleen during *C. irritans* infection, whereas the *N. seriolae* dataset exhibited more tissue- and stage-dependent expression patterns, marked by pronounced *fbxo2* induction in the liver and relatively high *canx* expression in the kidney and spleen (Figure S7). In addition, the selected PRR-related genes *nod2*, *tlr5*, and *tlr7* showed distinct expression patterns across the three pathogen infection models, and their correlations with the candidate genes varied accordingly (Figure S8).

RT-qPCR was conducted to further verify the molecular response under varied pathogenic pressures. We evaluated the temporal dynamics during *N. seriolae* infection over a 24 h period (Figure 9D). The results revealed that all six genes responded significantly to the infection with diverse peak times: *rnd3* and *canx* were upregulated, where *rnd3* showed a significant change at 9 and 12 h. Angptl4 displayed an early response peak at 6 h followed by a gradual decline, while *vdr* and *cbx7* showed statistically significant fluctuations. In the *P. damselae* infection model (Figure 9E), *canx* and rnd3 were significantly upregulated (*p* < 0.05). Conversely, the expression levels of *vdr*, *angptl4*, and *fbxo2* were significantly downregulated.

## 4. Discussion

### 4.1. Identification of Cross-Pathogen Biomarkers and Divergent Immune Regulatory Strategies

Multiple pathogen infections severely constrain the sustainable development of Silver Pomfret aquaculture [1,2]. A major bottleneck in current disease management is the reliance on pathogen-specific diagnostics, which fail to address the complex reality of overlapping pathogenic pressures [14]. Most previous transcriptomic studies in fish have focused on individual infection models [3], which are useful for describing pathogen-specific responses but may overlook host factors that are consistently involved across different infectious conditions. In the present study, we identified a concise panel of cross-pathogen-responsive candidate biomarkers, including *vdr*, *angptl4*, *fbxo2*, *cbx7*, *canx*, and *rnd3*. These genes were shared among infections caused by three pathogen infections, suggesting a context-dependent host response under diverse pathogenic challenges.

The significance of these shared genes likely lies not only in uniform regulation across all infections, but also in the different pathological settings in which they are engaged. In Silver Pomfret, host responses appear to be shaped by the need to balance pathogen control with the preservation of tissue integrity, and the relative emphasis placed on these processes may vary with pathogen type and infection mode. Unlike classical immune markers, the present panel was derived from cross-model transcriptomic convergence and network relevance. Classical immune genes may remain important in individual infections but may not emerge as stable markers across heterogeneous infectious contexts. Although *N. seriolae* and *P. damselae* are both bacterial pathogens, the diseases they cause differ substantially in progression and tissue involvement. *N. seriolae* is typically associated with chronic granulomatous infection and prolonged intracellular persistence, which may require sustained immune activation over an extended period [3]. By contrast, *P. damselae* infection is often characterized by acute septicemic progression and rapid tissue injury, conditions that may impose stronger immediate inflammatory stress on the host [1]. *C. irritans* differs from both bacterial models in that it primarily infects the skin and gills, causing localized epithelial and mucosal damage rather than early systemic invasion [2]. These biological differences provide an important context for interpreting the distinct enrichment patterns and immune cell signatures observed in this study.

Studying the relationship between pathogen-associated molecular patterns (PAMPs) and pattern-recognition receptors (PRRs) is a useful way to interpret these differences. Different pathogens expose the host to different dominant molecular cues, and these are expected to bias the initial recognition phase and, consequently, the downstream transcriptional program [15]. In our datasets, the expression patterns of *nod2*, *tlr5*, and *tlr7*, together with their correlations with the candidate genes, support this view. The two bacterial infections showed stronger and more coordinated associations between PRR-related genes and the candidate panel, whereas the parasitic model showed a more selective and context-dependent pattern. This suggests that the identified six-gene panel is not acting as a direct surrogate for any single PRR pathway; rather, it may reflect downstream host programs engaged after different PAMP–PRR inputs have been integrated. In this sense, upstream recognition appears pathogen-dependent, whereas the shared candidate genes may capture a partially convergent stress-adaptive and immune-regulatory output.

This interpretation is also consistent with the immune infiltration profiles. The two bacterial infections were associated with broader systemic immune perturbation than the parasitic model, although their immune characteristics were not identical. In the *N. seriolae* model, the concurrent enrichment of cytotoxic T cell, exhausted T cell-like, Th17-like, and Treg-like signatures, together with associations with neutrophil, monocyte, and dendritic cell populations, may indicate a coordinated immune state involving both sustained cellular activation and compensatory immunoregulation. This pattern is consistent with the chronic nature of nocardiosis, in which persistent intracellular infection is likely to require prolonged antigen presentation, myeloid-cell participation, and T cell-mediated responses [6]. In contrast, the immune profile of *P. damselae* infection was characterized by marked changes in neutrophil, B cell, effector memory, and Treg-like signatures, accompanied by decreases in several T cell-related populations, suggesting a more destabilized systemic immune state during acute infection [7]. Thus, while both bacterial pathogens induced strong host responses, the underlying immune organization appeared to differ between chronic intracellular infection and acute septicemic challenge. The response to *C. irritans* appeared to follow a different immunological pattern. Compared with the bacterial models, the parasitic infection produced more moderate systemic immune alterations, with relatively greater prominence of Th2- and Treg-like-associated signatures. This pattern may suggest a more regulatory and tissue-protective immune state during parasitic infestation, particularly at barrier surfaces. Such an interpretation is biologically plausible in Silver Pomfret. This species possesses small and easily detached scales, which may make the body surface relatively vulnerable to physical disruption and secondary microbial invasion once the mucus–epithelial barrier is damaged [16,17]. Under these conditions, excessive local inflammation could further aggravate tissue injury and compromise barrier integrity. The host response to *C. irritans* may therefore favor immune regulation and tissue maintenance over strong systemic inflammatory activation, thereby limiting self-inflicted damage at already compromised epithelial sites.

The functional enrichment results were broadly consistent with these immune infiltration patterns and suggest that the distinct immune landscapes were accompanied by different cellular priorities. In the bacterial models, the enrichment of lysosomal, proteasomal, and related stress-processing pathways may reflect increased demands for pathogen degradation, protein turnover, and intracellular homeostasis under systemic infectious stress. Such processes are likely to be particularly important during bacterial invasion, when inflammatory activation, tissue injury, and cellular stress occur simultaneously. In contrast, the parasitic model showed relative suppression of several intracellular processing and defense-associated pathways, suggesting that the response to *C. irritans* does not simply involve amplification of the same systemic programs observed during bacterial infection. Instead, it may involve a shift toward a more restrained response that reduces metabolically costly or potentially tissue-damaging immune activation while preserving epithelial stability. This pattern could represent a host adaptive strategy under localized barrier injury, although the possibility of parasite-mediated modulation of host immune and metabolic processes cannot be excluded.

Despite these differences, the three infection models shared enrichment in many common pathways, suggesting potential convergence on common host processes related to cellular stress control, metabolic adjustment, and tissue repair. Within this framework, the six candidate genes identified here may represent molecular links between conserved stress-adaptive processes and pathogen-dependent immune responses. For example, *canx* is functionally associated with endoplasmic reticulum protein folding and cellular stress regulation, whereas *angptl4* has been implicated in lipid metabolism, inflammatory modulation, and tissue repair [18,19]. Their identification across all three infection models suggests that these genes may participate in core host processes repeatedly engaged during infection, while their differential expression patterns further imply that they may contribute differently depending on the pathological context.

### 4.2. Regulatory Mechanisms and Functional Coordination of Candidate Biomarkers in the Immune Responses of Silver Pomfret

The cross-pathogen response signature in *P. argenteus* appears to involve a coordinated regulatory network that links upstream transcriptional modulation with downstream structural and metabolic effector functions, integrating upstream transcriptional modulation with downstream structural and metabolic effector functions. Upstream of this process, the transcription factor CTCF may act as a primary modulator of chromatin organization and cell cycle progression [20]. The stable temporal expression of *ctcf* aligns with our previous transcriptomic enrichment results, and may help provide the genomic plasticity required to fuel the rapid proliferation of immune cells [21]. Concurrently, the miR-17a/20ab/93 cluster may serve as an important post-transcriptional regulator. As a member of the miR-17/92 family [22], this miRNA cluster has been reported to influence the differentiation fate of effector T cells and to help maintain immune tolerance [23,24]. This regulatory mechanism is consistent with our immune infiltration analysis, which showed marked changes in activated T cells and neutrophil populations. In addition, the pathogen-specific correlation patterns among the six candidate genes further suggest that the common diagnostic signature identified in this study is probably supported by a flexible transcriptional coordination framework rather than by a completely fixed regulatory module. This is consistent with the GSVA and perturbation analysis, which showed that the six-gene panel retained cross-pathogen discriminatory value, whereas the relative contribution of individual genes varied across infection models. While these upstream mechanisms may support early immune defenses, the prevention of host tissue damage relies heavily on epigenetic and transcriptional suppressors to maintain immune tolerance [24,25]. Our qPCR results revealed a profound late-phase downregulation of *vdr* and *cbx7* across the bacterial infection models. Given that *vdr* and *cbx7* are implicated in preventing excessive inflammatory cascades and maintaining epigenetic suppression of pro-inflammatory loci [26,27], their severe suppression may indicate a progressive loss of immune tolerance. This interpretation is also supported by the positive correlation observed between *angptl4* and *vdr* in the *P. damselae* infection, as it implies that metabolic adaptation and anti-inflammatory regulation may be transcriptionally coupled during bacterial injury. Likewise, the relatively positive correlation between *rnd3* and *cbx7* suggests that cytoskeletal repair responses may remain linked to epigenetic or transcriptional restraint mechanisms under this condition. Consistent with this view, perturbation of *cbx7* also produced substantial rollback in both the *C. irritans* and *N. seriolae* cohorts, suggesting that epigenetic restraint may contribute materially to signature stability under distinct infection contexts. This regulatory imbalance may contribute to the severe immunopathology and high mortality observed in Silver Pomfret during prolonged *P. damselae* and *N. seriolae* infections.

At the cellular effector level, our PPI analysis and temporal expression profiles suggest a functional coupling between *canx* and *rnd3*, which may collectively coordinate intracellular proteostasis and tissue structural repair. Rather than direct physical binding, the interaction between the endoplasmic reticulum (ER) chaperone Canx and the cytoskeletal regulator Rnd3 is more likely to represent an indirect functional relationship supported by shared or intermediate signaling components [18,28], specifically PHLPP, Tnks, and the transcription factor Elk1, which may collectively coordinate cytoplasmic phosphorylation and nuclear gene activation to integrate the protein folding quality control of Canx with the mechanical barrier stabilization driven by Rnd3 [29,30,31]. During bacterial invasion, *canx* showed a sustained upregulation in the kidney, liver, and spleen. Furthermore, the qPCR validation further supported the marked responsiveness of *canx*. This pattern suggests that *canx* may help alleviate pathogen-induced ER stress by facilitating immune-related protein folding in Silver Pomfret. Consistent with this, perturbation of canx produced the largest rollback in the *P. damselae* cohort, indicating a relatively strong contribution to the acute bacterial injury-associated signature. Within this framework, rapid induction of *rnd3* in the kidney during acute *P. damselae* infection may support actin cytoskeleton dynamics, immune cell migration, and repair of disrupted tight junctions [32]. Given the high susceptibility of Silver Pomfret to bacterial infection due to its fragile and deciduous scales [16], the coordinated activity of Canx, Rnd3, and their putative intermediate regulators may represent a coupled response that both alleviates intracellular protein stress and helps preserve barrier integrity during bacterial injury, particularly under infections associated with skin ulceration and tissue necrosis, such as those caused by *P. damselae* and *N. seriolae*.

Parallel to this structurally coupled network, *angptl4* appears to function as another important node that reflects the immunometabolic dimension of infection. The mounting of a systemic immune response imposes tremendous energetic demands on the host [33]. Recognized as a core mediator of lipid metabolism, *angptl4* may participate in the rapid metabolic reprogramming required to support acute-phase inflammation [19]. In *N. seriolae* infection, the qPCR results demonstrated a rapid but transient surge of *angptl4* expression within the first 6 h after infection, followed by a precipitous decline. This rapid induction implies an immediate redirection of lipid utilization to meet the high physiological demands required for the initial clearance of this intracellular pathogen. In contrast, the early stage of *P. damselae* infection was characterized by a significant downregulation of *angptl4*, although transcriptomic analysis revealed a gradual elevation of its expression as the disease progressed into later stages. These divergent expression patterns likely reflect the different pathological impacts and infection strategies of the two bacteria. This context dependence is also consistent with the perturbation analysis, in which angptl4 exerted a stronger effect in *N. seriolae* than in *P. damselae*, suggesting that its contribution to the shared signature may be more prominent in the intracellular infection setting than in the acute septicemic model. The early metabolic response to *N. seriolae* may be related either to enhanced macrophage-associated activity or to altered lipid allocation during the initial intracellular host–pathogen interaction. Conversely, the initial suppression during *P. damselae* infection might be attributed to the acute toxemia and rapid tissue necrosis caused by this pathogen [3], which could temporarily overwhelm the host metabolic machinery before a compensatory recruitment of *angptl4* occurs during the systemic phase of the disease. The correlation analysis provided additional support for this context-dependent role of Angptl4. In the *C. irritans* model, *angptl4* showed positive correlations with most candidate genes but a clear negative correlation with *fbxo2*, suggesting that acute metabolic redistribution during parasitic stress may be dissociated from ubiquitin-dependent proteostatic turnover. In contrast, the positive correlation between *fbxo2* and angptl4 under the *N. seriolae* infection implies a stronger correlation between metabolic adaptation and protein quality-control processes during intracellular bacterial infection. However, the eventual reduction in *angptl4* expression in both models, combined with the significant downregulation of the ubiquitin ligase component *fbxo2* [34], suggests that Silver Pomfret may experience impaired metabolic and proteostatic capacity at later stages of infection. The suppression of *fbxo2*-mediated protein degradation likely serves as a compensatory energy saving mechanism to preserve essential cellular components when the lipid mobilization driven by angptl4 can no longer meet the energetic costs of the systemic immune response [35].

While the functional coordination of these biomarkers was most clearly supported by transcriptomic analysis and qPCR in the acute extracellular damage model (*P. damselae*) and the chronic intracellular infection model (*N. seriolae*), their distinct roles also provide a useful basis for discussing host responses to the parasitic ciliate *C. irritans*. Characterized by intense localized mechanical disruption of the mucosal epithelium [3], *C. irritans* infestation would be expected to place strong demands on tissue protection and repair, potentially increasing the importance of the Canx-PHLPP/Tnks-Rnd3 structural repair axis to prevent massive secondary bacterial colonization. Furthermore, the localized inflammatory burden would rapidly deplete metabolic reserves, heavily relying on *angptl4*-independent energy redistribution [36,37]. Although direct in vivo validation for the parasitic model was restricted in the current study due to the exhaustion of experimental samples, the repeated identification of these candidate genes across comparative transcriptomics, network analysis, and qPCR validation supports their potential relevance as candidate infection-responsive markers. Future studies incorporating *C. irritans* challenge models will be required to validate the temporal dynamics of these biomarkers, thereby optimizing their application in the multi-pathogen surveillance of Silver Pomfret aquaculture.

Nevertheless, several limitations should be considered. This study integrated previously published transcriptomic datasets, meaning that technical heterogeneity from sequencing, sample processing, and analytical workflows cannot be excluded. Limited and uneven sample representation across infection models may also have reduced statistical power and affected model stability and generalizability; therefore, the LOPO analysis should be viewed as demonstrating cross-pathogen transferability rather than formal external validation. Because only three pathogens were included, the six-gene panel should be considered a provisional signature in Silver Pomfret, with uncertain applicability to other fish species. Also, incomplete genome annotation and limited cell-type marker mapping may restrict biological interpretation. Accordingly, ssGSEA-based immune inference provides only indirect estimates rather than validated immune cell states. Although permutation importance and LASSO reduced model dependence, gene contributions remain method-sensitive. Therefore, these genes should still be regarded as candidate biomarkers requiring further validation in larger cohorts, under field-relevant conditions, and through single-cell, cross-tissue, and functional studies.

## 5. Conclusions

This study identified a six-gene biomarker panel in Silver Pomfret that responded across both bacterial and parasitic infection models. Integrating machine learning, comparative transcriptomics, and qPCR validation, our results suggest that *canx*, *rnd3*, and *angptl4* may represent important components of the host response under diverse pathogenic challenges. Together, these biomarkers appear to reflect coordinated processes related to structural repair, metabolic adjustment, and immune cell dynamics during infection. Their reproducible infection-associated patterns support their potential utility as candidate markers for cross-pathogen disease surveillance in Silver Pomfret. Overall, these findings provide a preliminary basis for future evaluation of their potential as candidate markers in cross-pathogen disease monitoring in Silver Pomfret.

## Figures and Tables

**Table 1:** Premiers used for qPCR in this study.

Gene Name	Sense-Sequence (5′-3′)	Anti-Sequence (5′-3′)	Amplicon Length (bp)
*vdr*	TGGAGCCGCTGGTGAAGTTC	TGCTGTGCTCTGGCTGGAAG	300
*canx*	CCTCTCGTCCTCATCGTTGTCT	TCCTCCTCCTCTTCCTCCTTCA	122
*rnd3*	ATGCTGTTGGTTGGCTGTAAGT	GCTGTTCTCCGACTGCTGTG	165
*angptl4*	GGTGAAGGTGAAGGTGGAGGAG	GCTGGTCAATGCGTCTGTTCTG	185
*cbx7*	ACCTGACAGCAGTAGGCACAA	TTAGACGCTCCGATCCTGACTC	207
*fbxo2*	CCAGTGGAAGGTGGAGGACAT	TGTGATGGCAGGCTGAGCTT	169

## Data Availability

The transcriptomic data have been deposited in the NCBI database under accession number PRJNA884829, PRJNA948898, PRJNA975702, and PRJNA783750.

## Supplementary Materials

The following supporting information can be downloaded at: https://www.mdpi.com/article/10.3390/ani16101510/s1, Figure S1: Diagnostic performance evaluation of single gene and global machine learning models across three infection groups. (A, B, C) ROC evaluating the predictive accuracy within *Cryptocaryon irritans*, *Nocardia seriolae*, and *Photobacterium damselae* infection datasets respectively. Each structural panel illustrates the specific diagnostic capabilities of the candidate genes compared against the integrated global models. The predictive performances of both the RF and SVM algorithms are explicitly plotted for visual comparison. Specific area under the curve values are detailed within each individual graph to objectively quantify the diagnostic sensitivity and specificity. Shaded regions beneath the global model curves are incorporated to visually emphasize the robust comprehensive predictive power achieved when all individual candidate genes are mathematically combined into a single diagnostic framework. Figure S2: Additional validation of the six-gene diagnostic signature across infection models. (A) ROC curves for LOPO validation. In each round, two infection models were used as the training set and the remaining one as the test set, generating three training–test combinations. (B) Comparison of classification performance between the LASSO-logistic regression and Random Forest models. DeLong’s test was used to assess the difference between the two classifiers. (C) ROC analysis of the six-gene panel in an external low-temperature stress dataset. (D) Empirical null distribution of LOPO AUC values generated from 1000 repeated resampling tests. (E) Cross-validated permutation importance ranking of the six genes. Figure S3: Sankey plot of GO enrichment of the candidate genes. Figure S4: Phylogenetic and structural characterization of the candidate genes. (A–F) Phylogenetic trees of Vdr, Rnd3, Fbxo2, Cbx7, Canx, Angptl4. (G) conserved domains of candidate gens: Abbreviations Mm: *Mus musculus*, Hs: *Homo sapiens*, Lc: *Larimichthys crocea*, Dr: *Danio rerio*, Ta: *Thunnus albacares*, Tt: *Thunnus thynnus*, Tm: *Thunnus maccoyii*, and Pa: *Pampus argenteus*. Figure S5: Cross-strategy consistency of virtual perturbation results. (A) Heatmap of the concordance of gene contribution rankings across four perturbation strategies (median, mean, random control draw, and conditional Gaussian) in the three cohorts. (B) Rank-tile plot showing gene importance orders derived from rollback analysis across perturbation strategies in the three cohorts. Figure S6: 3D structure of the hub gene predicted in Swiss-model. Sky blue represents the alpha helix, light blue stands for the extended strand, and light purple is for the beta turn and sheet, while pink is for the random coil. Figure S7: Tissue expression patterns of the six candidate genes in different infection settings. Left panel is the Eexpression levels of genes in gill (G), kidney (K), and spleen (S) tissues under control (C) and treatment (T) conditions. While right panel shows the expression levels of the same six genes in kidney (K), liver (L), and spleen (S) tissues under control (C), 15 days post-infection (H), and 5 days post-infection (L) conditions. Figure S8: Expression patterns and correlation analysis of PRR-related genes across three pathogen infection models in Silver Pomfret. (A-C) Expression patterns in three groups. (D-F) Correlation relationship in three groups. Table S1. Sample counts of the transcriptomes. Table S2. Websites using in this study. Table S3. Gene Ontology and KEGG pathway annotations of the target genes. Table S4. Characters of target genes. Table S5. Second structure of Target genes. Table S6. Signatures of perturbation. Table S7. Details of the significance test of in silico analysis under different strategies. Table S8. Predicted miRNAs of targeted genes.

## References

[1] Li Y., Hu J., Zhang Y., Yan K., Wang X., Zhou S., Xu S., Yan X., Wang Y. (2024). Complement C1q Is Involved in the Activation of Membrane Attack Complexes, Regulation of Bacterial Infectious Inflammation, and Apoptosis through Overexpression in Primary Cells of Silver Pomfret (*Pampus Argenteus*) in Vitro. Int. J. Biol. Macromol..

[2] Wang G., Hu J., Zhang M., Zhang Y., Li Y., Jiang H., Wang X., Zhu J., Xu S., Wang Y. (2023). Histopathology, Immunoenzyme Activity and Transcriptome Analysis of Immune Response in Silver Pomfret Infected by Cryptokaryon (*Cryptorchidism Irritant*). Fish Shellfish. Immunol..

[3] Zhang Y., Hu J., Yan K., Yuan F., Li Y., Zhang M., Li Y., Huang X., Tang J., Wang D. (2024). Immune Response of Silver Pomfret (*Pampus Argenteus*) to *Photobacterium Damselae* Subsp. *Damselae*: Virulence Factors Might Induce Immune Escape by Damaging Phagosome. Aquaculture.

[4] Li X., Yuan L., Huang K., Chen S., Zhou S., Ma R., Jiang J., Xie J. (2024). Isolation and Characterization of a Bacteriophage for Biological Control of *Photobacterium Damselae* Subsp. *Damselae* in Silver Pomfret Mariculture. Aquaculture.

[5] Jerônimo G.T., Cruz M.G., Aguiar Bertaglia E.D., Furtado W.E., Martins M.L. (2022). Fish Parasites Can Reflect Environmental Quality in Fish Farms. Rev. Aquac..

[6] Nawaz M., Gao T., Huang K., Gouife M., Chen S., Zhu S., Ma R., Jin S., Jiang J., Xie J. (2022). Pathogenicity, Diagnosis, Prevention Strategies and Immune Response of Bacterium Nocardia Seriolae: A Critical Review. Aquac. Res..

[7] Tao Z., Shen C., Zhou S.-M., Yang N., Wang G.-L., Wang Y.-J., Xu S.-L. (2018). An Outbreak of *Photobacterium Damselae* Subsp. *Damselae* Infection in Cultured Silver Pomfret *Pampus Argenteus* in Eastern China. Aquaculture.

[8] Zhang Y., Hu J., Li Y., Yuan F., Yan K., Gu W., Zhang M., Li Y., Huang X., Zhang C. (2024). Immune Strategies of Silver Pomfret (*Pampus Argenteus*) Infected with *Nocardia Seriolae* at Different Infection Stages. Aquaculture.

[9] Zhang M., Hu J., Zhu J., Wang Y., Zhang Y., Li Y., Xu S., Yan X., Zhang D. (2022). Transcriptome, Antioxidant Enzymes and Histological Analysis Reveal Molecular Mechanisms Responsive to Long-Term Cold Stress in Silver Pomfret (*Pampus Argenteus*). Fish Shellfish. Immunol..

[10] Chen C., Chen H., Zhang Y., Thomas H.R., Frank M.H., He Y., Xia R. (2020). TBtools: An Integrative Toolkit Developed for Interactive Analyses of Big Biological Data. Mol. Plant.

[11] Xie J., Chen Y., Cai G., Cai R., Hu Z., Wang H. (2023). Tree Visualization By One Table (tvBOT): A Web Application for Visualizing, Modifying and Annotating Phylogenetic Trees. Nucleic Acids Res..

[12] Hu C., Zhang N., Hong Y., Tie R., Fan D., Lin A., Chen Y., Xiang L., Shao J. (2024). Single-Cell RNA Sequencing Unveils the Hidden Powers of Zebrafish Kidney for Generating Both Hematopoiesis and Adaptive Antiviral Immunity. eLife.

[13] Ji X., Tang J., Zhang J. (2022). Effects of Salt Stress on the Morphology, Growth and Physiological Parameters of *Juglansmicrocarpa* L. Seedlings. Plants.

[14] Patnaik A., Kayal T., Basu S. (2025). Polymicrobial Infections: A Comprehensive Review on Current Context, Diagnostic Bottlenecks and Future Directions. Acta Microbiol. Hell..

[15] Betancourt J.L., Rodríguez-Ramos T., Dixon B. (2024). Pattern Recognition Receptors in Crustacea: Immunological Roles under Environmental Stress. Front. Immunol..

[16] Tang J., Zhou S., Hu J.B., Wang X.B., Wang G.L., Jiang H., Yan X.J. (2023). Early covery of squmation and the development of primary scales for *Pampus argenteus*. ACTA Hydrobiol. Sin..

[17] Hu J., Zhang Y., Li Y., Li Y., Zhang M., Huang W., Xu S., Wang D., Wang X., Liu J. (2024). Two High Quality Chromosome-Scale Genome Assemblies of Female and Male Silver Pomfret (*Pampus Argenteus*). Sci. Data.

[18] Yan G., Li X., Zheng Z., Gao W., Chen C., Wang X., Cheng Z., Yu J., Zou G., Farooq M.Z. (2022). KAT7-Mediated CANX (Calnexin) Crotonylation Regulates Leucine-Stimulated MTORC1 Activity. Autophagy.

[19] Chaube B., Citrin K.M., Sahraei M., Singh A.K., de Urturi D.S., Ding W., Pierce R.W., Raaisa R., Cardone R., Kibbey R. (2023). Suppression of Angiopoietin-like 4 Reprograms Endothelial Cell Metabolism and Inhibits Angiogenesis. Nat. Commun..

[20] Sun X., Zhang J., Cao C. (2022). CTCF and Its Partners: Shaper of 3D Genome during Development. Genes.

[21] Arzate-Mejía R.G., Recillas-Targa F., Corces V.G. (2018). Developing in 3D: The Role of CTCF in Cell Differentiation. Development.

[22] Meenhuis A., van Veelen P.A., de Looper H., van Boxtel N., van den Berge I.J., Sun S.M., Taskesen E., Stern P., de Ru A.H., van Adrichem A.J. (2011). *MiR-17/20/93/106* Promote Hematopoietic Cell Expansion by Targeting Sequestosome 1–Regulated Pathways in Mice. Blood.

[23] Mogilyansky E., Rigoutsos I. (2013). The miR-17/92 Cluster: A Comprehensive Update on Its Genomics, Genetics, Functions and Increasingly Important and Numerous Roles in Health and Disease. Cell Death Differ..

[24] Izreig S., Samborska B., Johnson R.M., Sergushichev A., Ma E.H., Lussier C., Loginicheva E., Donayo A.O., Poffenberger M.C., Sagan S.M. (2016). The miR-17∼92 microRNA Cluster Is a Global Regulator of Tumor Metabolism. Cell Rep..

[25] Zhang L., Xiao X., Arnold P.R., Li X.C. (2019). Transcriptional and Epigenetic Regulation of Immune Tolerance: Roles of the NF-κB Family Members. Cell Mol. Immunol..

[26] Sirohi K., Sripada A., Verma M., Varma R., Liu S., Yadav S., Sahu A., Manka L., Guntur V.P., Gorska M.M. (2026). CBX7 Functions as a Methylation-Dependent Inducer of Gene Transcription and Regulator of Cytosolic Signaling in Lymphoid Cells. Sci. Adv..

[27] Froicu M., Cantorna M.T. (2007). Vitamin D and the Vitamin D Receptor Are Critical for Control of the Innate Immune Response to Colonic Injury. BMC Immunol..

[28] Pacary E., Azzarelli R., Guillemot F. (2013). Rnd3 Coordinates Early Steps of Cortical Neurogenesis through Actin-Dependent and -Independent Mechanisms. Nat. Commun..

[29] Grzechnik A.T., Newton A.C. (2016). PHLPPing through History: A Decade in the Life of PHLPP Phosphatases. Biochem. Soc. Trans..

[30] Huang S.-M.A., Mishina Y.M., Liu S. (2009). Tankyrase Inhibition Stabilizes Axin and Antagonizes Wnt Signalling. Nature.

[31] Kasza A. (2013). Signal-Dependent Elk-1 Target Genes Involved in Transcript Processing and Cell Migration. Biochim. Et Biophys. Acta (BBA)-Gene Regul. Mech..

[32] Jie W., Andrade K.C., Lin X., Yang X., Yue X., Chang J. (2016). Pathophysiological Functions of Rnd3/RhoE. Compr. Physiol..

[33] Wensveen F.M., Šestan M., Polić B. (2024). The Immunology of Sickness Metabolism. Cell Mol. Immunol..

[34] Skaar J.R., Pagan J.K., Pagano M. (2013). Mechanisms and Function of Substrate Recruitment by F-Box Proteins. Nat. Rev. Mol. Cell Biol..

[35] Glenn K.A., Nelson R.F., Wen H.M., Mallinger A.J., Paulson H.L. (2008). Diversity in Tissue Expression, Substrate Binding, and SCF Complex Formation for a Lectin Family of Ubiquitin Ligases*. J. Biol. Chem..

[36] Lichtenstein L., Mattijssen F., de Wit N.J., Georgiadi A., Hooiveld G.J., van der Meer R., He Y., Qi L., Köster A., Tamsma J.T. (2010). Angptl4 Protects against Severe Pro-Inflammatory Effects of Dietary Saturated Fat by Inhibiting Lipoprotein Lipase-Dependent Uptake of Fatty Acids in Mesenteric Lymph Node Macrophages. Cell Metab..

[37] Wang H., Ye J. (2015). Regulation of Energy Balance by Inflammation: Common Theme in Physiology and Pathology. Rev. Endocr. Metab. Disord..

