# Supplementary material for: Transcriptomic Analysis and Machine Learning Identify Cross-Pathogen Biomarkers for Bacterial and Parasitic Infections in Silver Pomfret (Pampus argenteus)
Source: Animals (Basel). 2026 May 14;16(10):1510. doi: 10.3390/ani16101510 (PMC13203390; doi:10.3390/ani16101510)
Supplement: Supplementary file 1 [file animals-16-01510-s001.zip › Supplementary Tables.pdf]

Supplementary Table captions

Table S1. Sample counts of the transcriptomes.

Table S2. Websites using in this study.

Table S3. Gene Ontology and KEGG pathway annotations of the target genes.

Table S4. Characters of target genes.

Table S5. Second structure of Target genes.

Table S6. Signatures of perturbation.

Table S7. Details of the significance test of in silico analysis under different strategies.

Table S8. Predicted miRNAs of targeted genes

Table. S1 Sample counts of the transcriptomes

Database	sample counts	n control	n infect
Photobacterium damselae subsp. damselae	45	9	36
Nocardia seriolae	18	9	9
Cryptocaryon irritans	27	9	18
Low temperature	27	9	18

Table. S2 Websites using in this study

Website	Link
Batch CD-Search	https://www.ncbi.nlm.nih.gov/Structure/bwrpsb/bwrpsb.cgi
Chiplot	https://www.chiplot.online/
SOPMA	https://npsa.lyon.inserm.fr/cgi-bin/npsa_automat.pl?page=/NPSA/npsa_sopma.html
ExPASy	https://web.expasy.org/protparam/
WoLF PSORT	https://wolfpsort.hgc.jp/
TMHMM 2.0	https://services.healthtech.dtu.dk/services/TMHMM-2.0/
SignalP 6.0	https://services.healthtech.dtu.dk/services/SignalP-6.0/
JASPAR	https://jaspar.elixir.no/
ChIP-Atlas	https://chip-atlas.org/
TargetScanFish 6.2	https://www.targetscan.org/fish_62/

Table. S3 Gene Ontology and KEGG pathway annotations of the target genes

Gene	GO ID	GO Term	Category	KEGG ID	KEGG Pathway
canx	GO:0051082	unfolded protein binding	MF	dre04141	Protein processing in endoplasmic reticulum
	GO:0030433	protein folding	BP	dre04612	Antigen processing and presentation
	GO:0034663	endoplasmic reticulum chaperone complex	CC	—	—
fbxo2	GO:0004842	ubiquitin-protein transferase activity	MF	dre04120	Ubiquitin mediated proteolysis
	GO:0019005	SCF ubiquitin ligase complex	CC	dre04141	Protein processing in endoplasmic reticulum
	GO:0031146	SCF-dependent proteasomal ubiquitin-dependent protein catabolic process	BP	—	—
rnd3	GO:0003924	GTPase activity	MF	—	—
	GO:0005525	GTP binding	MF	—	—
	GO:0030036	actin cytoskeleton organization	BP	—	—
	GO:0007264	small GTPase mediated signal transduction	BP	—	—

Gene	GO ID	GO Term	Category	KEGG ID	KEGG Pathway
angptl4	GO:0010469	regulation of receptor activity	BP	dre03320	PPAR signaling pathway
	GO:0030335	positive regulation of cell migration	BP	dre04979	Cholesterol metabolism
	GO:0008283	cell population proliferation	BP	—	—
vdr	GO:0003700	DNA-binding transcription factor activity	MF	dre04961	Endocrine and other factor-regulated calcium reabsorption
	GO:0005499	vitamin D binding	MF	dre04978	Mineral absorption
	GO:0045944	positive regulation of transcription by RNA polymerase II	BP	dre05152	Tuberculosis
	—	—	—	dre05207	Chemical carcinogenesis – receptor activation
cbx7	GO:0003682	chromatin binding	MF	—	—
	GO:0035519	histone H2A K119 ubiquitination	BP	—	—
	GO:0035102	PRC1 complex	CC	—	—
	GO:0006333	chromatin assembly or disassembly	BP	—	—

Table. S4 Characters of target genes

Gene name	Putative CDS (bp)	Size (aa)	Molecular weight (Da)	pI	Instability index(%)	Signal Peptide	Aliphatic index(%)	subcellular localization	transmembrane domain	Ext. coefficient	Grand average of hydropathicity
Vdr	687	228	25791.59	5.35	54.33	No	96.23	cytoplasmic and nuclear	0	14565	-0.21
Canx	1713	570	64035.36	4.32	41.17	Yes	66.95	Endoplasmic Reticulum	2	110850	-0.664
Rnd3	942	313	34824.71	9.4	59.26	No	70.42	extracellular	0	30660	-0.54
Angptl4	744	247	28392.48	8.79	52.76	Yes	87.98	extracellular	0	6990	-0.821
Cbx7	873	290	31692.7	4.96	65.1	No	55.1	nuclear	0	22710	-0.966
Fbxo2	261	86	9856.97	4.09	47.3	No	75.93	mitochondrion	0	18115	-0.476

Table. S5 Second structure of Target genes

Gene name	Alpha helix (%)	Beta bridge (%)	Beta turn (%)	Random coil (%)	Extended strand (%)
Vdr	54.82	0.00	0.00	37.72	7.46
Canx	20.70	0.00	0.00	67.89	11.40
Rnd3	24.6	0.00	0.00	62.94	12.46
Angptl4	87.04	0.00	0.00	10.53	2.43
Cbx7	20.69	0.00	0.00	70.34	8.97
Fbxo2	18.6	0.00	0.00	58.14	23.26

Table. S6 Signatures of perturbation

Cohort	gene	baseline_auc	ko_auc	delta_auc	ko_p	ko_p_label	signature_diff_before	signature_diff_after	signature_rollback_pct
P. damselae	ANGPTL4	0.787	0.728	-0.059	0.035	*P* = 0.0354	0.397	0.406	-2.304
P. damselae	CANX	0.787	0.642	-0.145	0.200	*P* = 0.2004	0.397	0.227	42.886
P. damselae	CBX7	0.787	0.676	-0.111	0.110	*P* = 0.1099	0.397	0.309	22.162
P. damselae	FBXO2	0.787	0.704	-0.084	0.062	*P* = 0.0623	0.397	0.401	-1.035
P. damselae	RND3	0.787	0.667	-0.121	0.131	*P* = 0.1307	0.397	0.274	31.038
P. damselae	VDR	0.787	0.744	-0.044	0.024	*P* = 0.0240	0.397	0.393	0.933
C. irritans	ANGPTL4	1.000	0.926	-0.074	0.001	*P* < 0.01	-0.668	-0.444	33.514
C. irritans	CANX	1.000	0.926	-0.074	0.001	*P* < 0.01	-0.668	-0.442	33.766
C. irritans	CBX7	1.000	0.889	-0.111	0.004	*P* < 0.01	-0.668	-0.422	36.731
C. irritans	FBXO2	1.000	1.000	0.000	0.000	*P* < 0.001	-0.668	-0.649	2.821
C. irritans	RND3	1.000	0.926	-0.074	0.001	*P* < 0.01	-0.668	-0.453	32.094
C. irritans	VDR	1.000	0.963	-0.037	0.000	*P* < 0.001	-0.668	-0.484	27.557
N. seriolae	ANGPTL4	0.764	0.488	-0.277	0.940	*P* = 0.9397	0.287	0.066	77.204
N. seriolae	CANX	0.764	0.728	-0.036	0.059	*P* = 0.0595	0.287	0.320	-11.447
N. seriolae	CBX7	0.764	0.580	-0.184	0.527	*P* = 0.5275	0.287	0.169	41.185
N. seriolae	FBXO2	0.764	0.667	-0.098	0.176	*P* = 0.1763	0.287	0.199	30.892
N. seriolae	RND3	0.764	0.722	-0.042	0.067	*P* = 0.0672	0.287	0.319	-10.893
N. seriolae	VDR	0.764	0.722	-0.042	0.067	*P* = 0.0672	0.287	0.358	-24.566

Table. S7 Details of the significance test of in silico analysis under different strategies

Cohort	Strategy	KO_gene	delta_auc_p_empirical	delta_auc_stability	rollback_p_empirical	rollback_stability
P. damselae	median	CBX7	0.07	0.97	0.03	0.99
P. damselae	median	VDR	0.84	0.39	0.72	0.36
P. damselae	median	FBXO2	0.32	0.84	0.50	0.75
P. damselae	median	RND3	0.09	0.95	0.03	0.99
P. damselae	median	CANX	0.01	0.99	0.08	0.96
P. damselae	median	ANGPTL4	0.62	0.29	0.36	0.18
P. damselae	mean	CBX7	0.61	0.70	0.65	0.68
P. damselae	mean	VDR	0.26	0.11	0.14	0.07
P. damselae	mean	FBXO2	1.00	0.49	0.61	0.30
P. damselae	mean	RND3	0.04	0.98	0.02	0.99
P. damselae	mean	CANX	0.00	1.00	0.02	0.99
P. damselae	mean	ANGPTL4	0.38	0.16	0.10	0.05
P. damselae	random_control_draw	CBX7	0.04	0.98	0.02	0.99
P. damselae	random_control_draw	VDR	0.74	0.34	0.67	0.33
P. damselae	random_control_draw	FBXO2	0.34	0.83	0.20	0.90
P. damselae	random_control_draw	RND3	0.04	0.98	0.03	0.98
P. damselae	random_control_draw	CANX	0.00	1.00	0.08	0.96
P. damselae	random_control_draw	ANGPTL4	0.53	0.23	0.20	0.10
P. damselae	conditional_gaussian	CBX7	0.22	0.89	0.18	0.91
P. damselae	conditional_gaussian	VDR	0.95	0.46	0.90	0.45
P. damselae	conditional_gaussian	FBXO2	0.46	0.77	0.49	0.75
P. damselae	conditional_gaussian	RND3	0.35	0.82	0.08	0.96
P. damselae	conditional_gaussian	CANX	0.14	0.93	0.24	0.88
P. damselae	conditional_gaussian	ANGPTL4	0.78	0.37	0.71	0.36
C. irritans	median	CBX7	0.40	0.00	0.00	1.00

Cohort	Strategy	KO_gene	delta_auc_p_empirical	delta_auc_stability	rollback_p_empirical	rollback_stability
C. irritans	median	VDR	0.58	0.00	0.00	1.00
C. irritans	median	FBXO2	1.00	0.04	0.71	0.65
C. irritans	median	RND3	0.49	0.00	0.00	1.00
C. irritans	median	CANX	0.39	0.00	0.00	1.00
C. irritans	median	ANGPTL4	0.52	0.00	0.04	0.98
C. irritans	mean	CBX7	0.38	0.00	0.00	1.00
C. irritans	mean	VDR	0.34	0.00	0.00	1.00
C. irritans	mean	FBXO2	1.00	0.03	0.41	0.79
C. irritans	mean	RND3	0.28	0.00	0.00	1.00
C. irritans	mean	CANX	0.29	0.00	0.00	1.00
C. irritans	mean	ANGPTL4	0.27	0.00	0.00	1.00
C. irritans	random_control_draw	CBX7	0.33	0.00	0.00	1.00
C. irritans	random_control_draw	VDR	0.34	0.00	0.00	1.00
C. irritans	random_control_draw	FBXO2	1.00	0.03	0.76	0.62
C. irritans	random_control_draw	RND3	0.51	0.00	0.01	1.00
C. irritans	random_control_draw	CANX	0.39	0.00	0.00	1.00
C. irritans	random_control_draw	ANGPTL4	0.50	0.01	0.00	1.00
C. irritans	conditional_gaussian	CBX7	0.80	0.00	0.01	1.00
C. irritans	conditional_gaussian	VDR	0.80	0.01	0.07	0.96
C. irritans	conditional_gaussian	FBXO2	1.00	0.03	0.62	0.69
C. irritans	conditional_gaussian	RND3	1.00	0.02	0.45	0.78
C. irritans	conditional_gaussian	CANX	0.95	0.02	0.12	0.94
C. irritans	conditional_gaussian	ANGPTL4	1.00	0.01	0.32	0.84
N. seriolae	median	CBX7	0.09	0.95	0.25	0.88
N. seriolae	median	VDR	0.66	0.29	0.28	0.14
N. seriolae	median	FBXO2	0.49	0.76	0.24	0.88
N. seriolae	median	RND3	1.00	0.47	0.70	0.35
N. seriolae	median	CANX	0.93	0.44	0.60	0.30
N. seriolae	median	ANGPTL4	0.00	1.00	0.33	0.83
N. seriolae	mean	CBX7	0.12	0.94	0.24	0.88
N. seriolae	mean	VDR	0.53	0.23	0.21	0.11
N. seriolae	mean	FBXO2	0.89	0.56	0.62	0.69
N. seriolae	mean	RND3	1.00	0.46	0.63	0.31
N. seriolae	mean	CANX	0.70	0.31	0.36	0.18
N. seriolae	mean	ANGPTL4	0.00	1.00	0.26	0.87
N. seriolae	random_control_draw	CBX7	0.11	0.94	0.27	0.86
N. seriolae	random_control_draw	VDR	0.63	0.26	0.31	0.15
N. seriolae	random_control_draw	FBXO2	0.46	0.77	0.28	0.86
N. seriolae	random_control_draw	RND3	1.00	0.45	0.71	0.35
N. seriolae	random_control_draw	CANX	0.89	0.40	0.45	0.22
N. seriolae	random_control_draw	ANGPTL4	0.01	1.00	0.34	0.83
N. seriolae	conditional_gaussian	CBX7	0.81	0.59	0.87	0.56
N. seriolae	conditional_gaussian	VDR	0.64	0.28	0.38	0.19
N. seriolae	conditional_gaussian	FBXO2	0.89	0.55	0.85	0.58
N. seriolae	conditional_gaussian	RND3	0.96	0.44	0.76	0.38
N. seriolae	conditional_gaussian	CANX	0.62	0.28	0.36	0.18
N. seriolae	conditional_gaussian	ANGPTL4	0.06	0.97	0.30	0.85

Table. S8 Predicted miRNAs of targeted genes

miRNA	Total_Sites	8mer	7mer_m8	7mer_1A	Total_Context_Score	Gene
miR-135	1	1	0	0	-0.17	*canx*
miR-27	2	0	1	1	-0.17	*canx*
miR-338	1	1	0	0	-0.16	*canx*
miR-140	1	0	1	0	-0.15	*canx*
miR-101	1	0	1	0	-0.12	*canx*
miR-141/200a	1	0	1	0	-0.12	*canx*
miR-1/206	1	0	1	0	-0.12	*canx*
miR-133	1	0	0	1	-0.09	*canx*
miR-129/722	1	0	1	0	-0.09	*canx*
miR-124	1	0	0	1	-0.08	*canx*
miR-17a/20ab/93	1	0	1	0	-0.07	*canx*
miR-7	1	0	0	1	-0.07	*canx*
miR-9	1	0	0	1	-0.05	*canx*
miR-128	1	0	0	1	-0.04	*canx*
miR-23	1	1	0	0	-0.04	*canx*
miR-181	1	1	0	0	-0.03	*canx*
miR-30	1	0	0	1	-0.02	*canx*
miR-216a	1	0	1	0	-0.02	*canx*
miR-731	1	0	0	1	-0.01	*canx*
miR-203	1	0	0	1	-0.01	*canx*
let-7	2	0	1	1	-0.29	*vdr*
miR-143	4	1	2	1	-0.27	*vdr*
miR-196	1	0	1	0	-0.24	*vdr*
miR-203	2	1	0	1	-0.2	*vdr*
miR-153	3	0	1	2	-0.17	*vdr*
miR-451	1	1	0	0	-0.15	*vdr*
miR-17a/20ab/93	1	0	1	0	-0.15	*vdr*
miR-15ab/16abc/457ab	3	0	3	0	-0.12	*vdr*
miR-205	1	0	0	1	-0.12	*vdr*
miR-34/34c	1	0	0	1	-0.11	*vdr*
miR-18	1	0	0	1	-0.11	*vdr*
miR-19	5	0	3	2	-0.09	*vdr*

miRNA	Total_Sites	8mer	7mer_m8	7mer_1A	Total_Context_Score	Gene
miR-29	1	0	1	0	-0.09	*vdr*
miR-430	1	1	0	0	-0.09	*vdr*
miR-455	1	0	1	0	-0.06	*vdr*
miR-133	1	1	0	0	-0.06	*vdr*
miR-216b	3	0	3	0	-0.06	*vdr*
miR-21	3	0	2	1	-0.05	*vdr*
miR-187	1	0	1	0	-0.05	*vdr*
miR-139	1	0	1	0	-0.05	*vdr*
miR-103/107	2	0	2	0	-0.04	*vdr*
miR-217	1	0	1	0	-0.03	*vdr*
miR-150	1	1	0	0	-0.03	*vdr*
miR-101	1	1	0	0	-0.03	*vdr*
miR-429/200bc	1	1	0	0	-0.03	*vdr*
miR-23	2	0	1	1	-0.03	*vdr*
miR-181	2	0	1	1	-0.03	*vdr*
miR-138	1	0	0	1	-0.03	*vdr*
miR-125	1	0	0	1	-0.03	*vdr*
miR-221/222	1	0	1	0	-0.02	*vdr*
miR-145	1	0	1	0	-0.02	*vdr*
miR-141/200a	1	0	1	0	-0.02	*vdr*
miR-1/206	1	0	0	1	-0.01	*vdr*
miR-204	1	0	0	1	-0.01	*vdr*
miR-7	1	0	0	1	-0.01	*vdr*
miR-214	1	0	0	1	-0.01	*vdr*
miR-144	1	0	0	1	-0.01	*vdr*
miR-25/92ab/363	1	0	0	1	-0.01	*vdr*
miR-133	1	0	1	0	-0.26	*cbx7*
miR-17a/20ab/93	1	0	1	0	-0.22	*cbx7*
miR-731	1	0	1	0	-0.17	*cbx7*
miR-21	1	0	1	0	-0.14	*cbx7*
miR-203	1	0	1	0	-0.12	*cbx7*
miR-140	1	0	0	1	-0.12	*cbx7*
miR-429/200bc	1	0	1	0	-0.11	*cbx7*
miR-7	1	0	0	1	-0.09	*cbx7*

miRNA	Total_Sites	8mer	7mer_m8	7mer_1A	Total_Context_Score	Gene
let-7	1	0	0	1	-0.09	*cbx7*
miR-30	1	0	0	1	-0.06	*cbx7*
miR-218	2	1	0	1	-0.34	*rnd3*
miR-455	1	1	0	0	-0.33	*rnd3*
miR-429/200bc	1	1	0	0	-0.28	*rnd3*
miR-27	2	1	0	1	-0.27	*rnd3*
miR-17a/20ab/93	3	1	2	0	-0.26	*rnd3*
miR-139	2	1	0	1	-0.23	*rnd3*
miR-130/301/454	1	0	1	0	-0.22	*rnd3*
miR-153	2	0	1	1	-0.22	*rnd3*
miR-21	4	2	1	1	-0.21	*rnd3*
miR-124	1	0	1	0	-0.2	*rnd3*
miR-128	2	0	1	1	-0.2	*rnd3*
miR-103/107	1	0	0	1	-0.18	*rnd3*
miR-217	1	0	1	0	-0.17	*rnd3*
miR-193	1	0	0	1	-0.16	*rnd3*
miR-338	1	0	0	1	-0.13	*rnd3*
miR-132/212	2	2	0	0	-0.12	*rnd3*
miR-9	1	1	0	0	-0.12	*rnd3*
miR-155/2194	3	1	1	1	-0.12	*rnd3*
miR-122	1	0	1	0	-0.11	*rnd3*
miR-30	2	1	1	0	-0.11	*rnd3*
miR-181	2	1	1	0	-0.09	*rnd3*
miR-205	1	0	0	1	-0.09	*rnd3*
miR-219	1	0	0	1	-0.09	*rnd3*
miR-145	1	0	0	1	-0.07	*rnd3*
miR-365	1	0	0	1	-0.06	*rnd3*
miR-22	1	0	0	1	-0.05	*rnd3*
miR-430	2	0	2	0	-0.04	*rnd3*
miR-375	1	0	0	1	-0.01	*rnd3*
miR-30	1	1	0	0	-0.28	*rnd3*
miR-143	1	1	0	0	-0.27	*rnd3*
miR-130/301/454	1	0	1	0	-0.24	*rnd3*
miR-19	1	0	1	0	-0.21	*rnd3*

miRNA	Total_Sites	8mer	7mer_m8	7mer_1A	Total_Context_Score	Gene
miR-193	1	0	0	1	-0.2	*rnd3*
miR-192	1	0	0	1	-0.19	*rnd3*
miR-223	1	0	0	1	-0.18	*rnd3*
miR-17a/20ab/93	1	0	1	0	-0.16	*rnd3*
miR-153	1	0	0	1	-0.08	*rnd3*
miR-137	1	0	0	1	-0.06	*rnd3*
miR-9	1	0	0	1	-0.05	*rnd3*
miR-129/722	1	0	0	1	-0.01	*rnd3*
miR-133	1	1	0	0	-0.35	*ANGPTL4*
miR-21	2	1	1	0	-0.33	*ANGPTL4*
miR-29	1	0	1	0	-0.23	*ANGPTL4*
miR-135	1	0	1	0	-0.23	*ANGPTL4*
miR-193	1	0	0	1	-0.16	*ANGPTL4*
miR-96	1	0	0	1	-0.14	*ANGPTL4*
miR-145	1	0	0	1	-0.14	*ANGPTL4*
miR-182	1	0	0	1	-0.14	*ANGPTL4*
miR-128	1	0	1	0	-0.14	*ANGPTL4*
miR-181	2	0	2	0	-0.13	*ANGPTL4*
miR-24	1	0	0	1	-0.12	*ANGPTL4*
miR-731	1	0	0	1	-0.12	*ANGPTL4*
miR-365	1	0	0	1	-0.11	*ANGPTL4*
miR-23	1	0	1	0	-0.1	*ANGPTL4*
miR-103/107	2	1	0	1	-0.59	*fbxo2*
miR-19	1	1	0	0	-0.38	*fbxo2*
let-7	1	0	1	0	-0.24	*fbxo2*
miR-731	1	0	1	0	-0.22	*fbxo2*
miR-196	1	0	1	0	-0.22	*fbxo2*
miR-23	1	0	1	0	-0.21	*fbxo2*
miR-140	1	0	0	1	-0.19	*fbxo2*
miR-17a/20ab/93	1	0	1	0	-0.17	*fbxo2*
miR-216b	1	0	1	0	-0.15	*fbxo2*
miR-153	1	0	0	1	-0.14	*fbxo2*
