# Supplementary figures and images for: Transcriptomic Analysis and Machine Learning Identify Cross-Pathogen Biomarkers for Bacterial and Parasitic Infections in Silver Pomfret (Pampus argenteus)
Source: Animals (Basel). 2026 May 14;16(10):1510. doi: 10.3390/ani16101510 (PMC13203390; doi:10.3390/ani16101510)

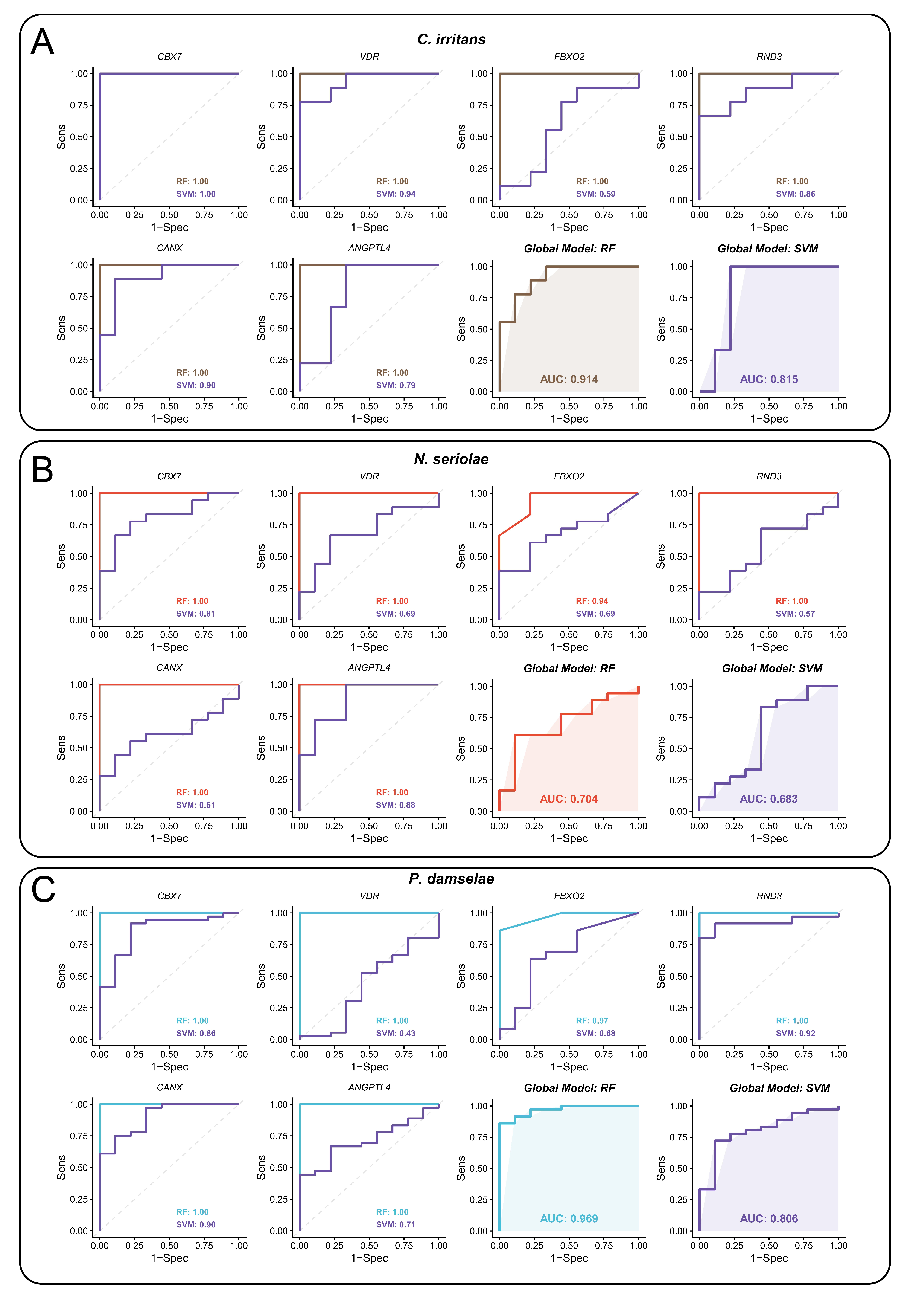

Supplement: Supplementary file 1 [file animals-16-01510-s001.zip › Supplementary Figures/Fig. S1.jpg]

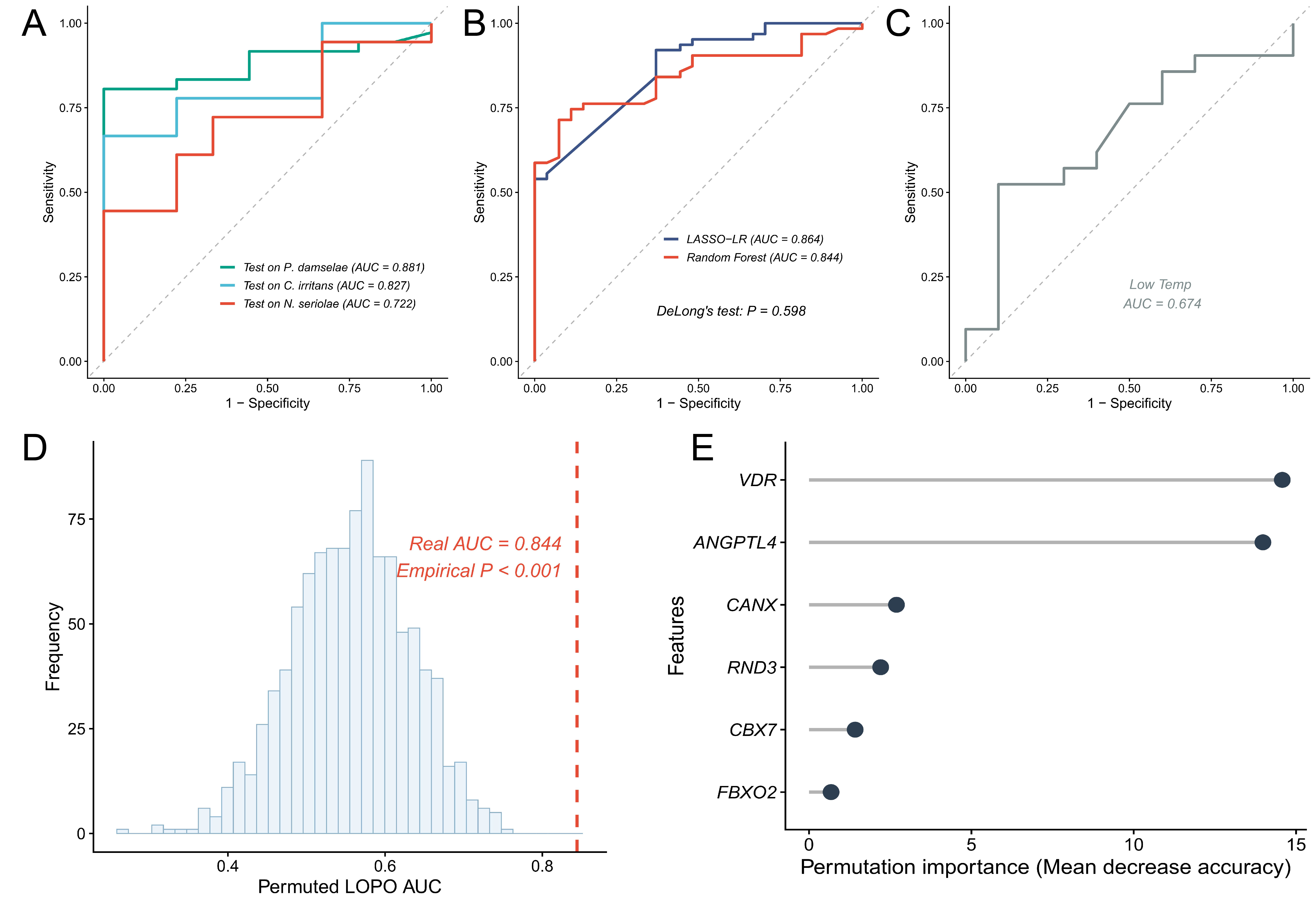

Supplement: Supplementary file 1 [file animals-16-01510-s001.zip › Supplementary Figures/Fig. S2.jpg]

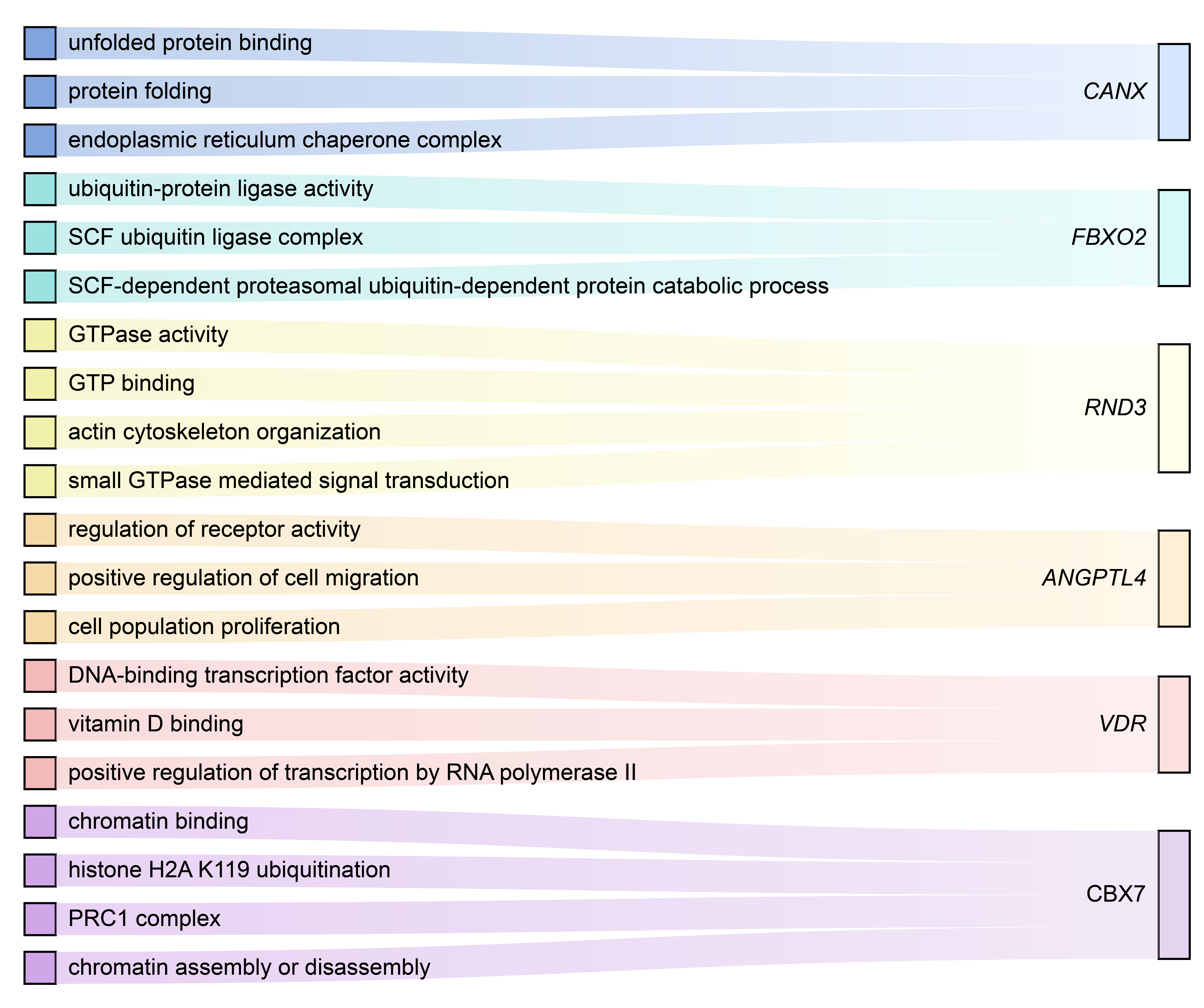

Supplement: Supplementary file 1 [file animals-16-01510-s001.zip › Supplementary Figures/Fig. S3.jpg]

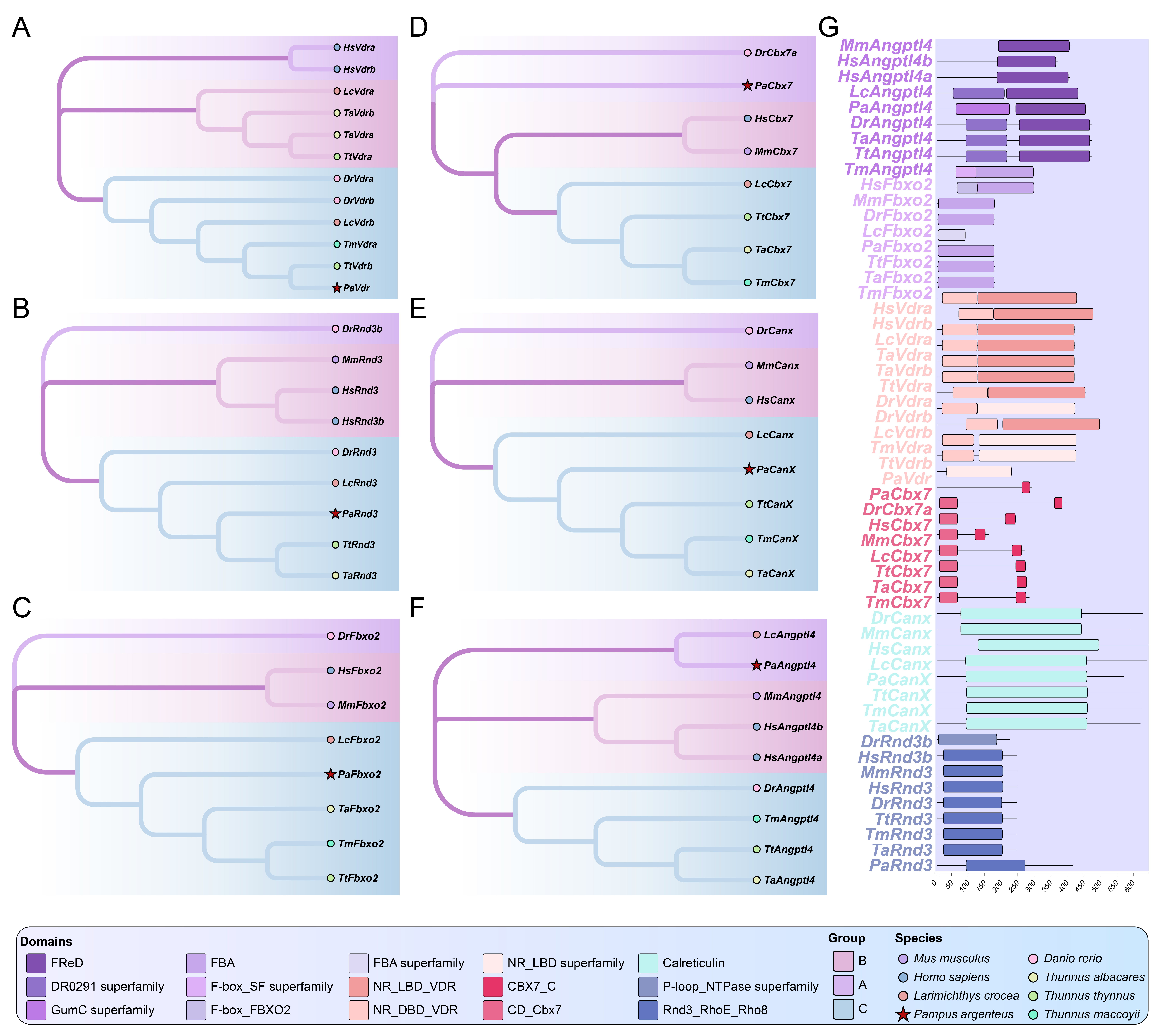

Supplement: Supplementary file 1 [file animals-16-01510-s001.zip › Supplementary Figures/Fig. S4.jpg]

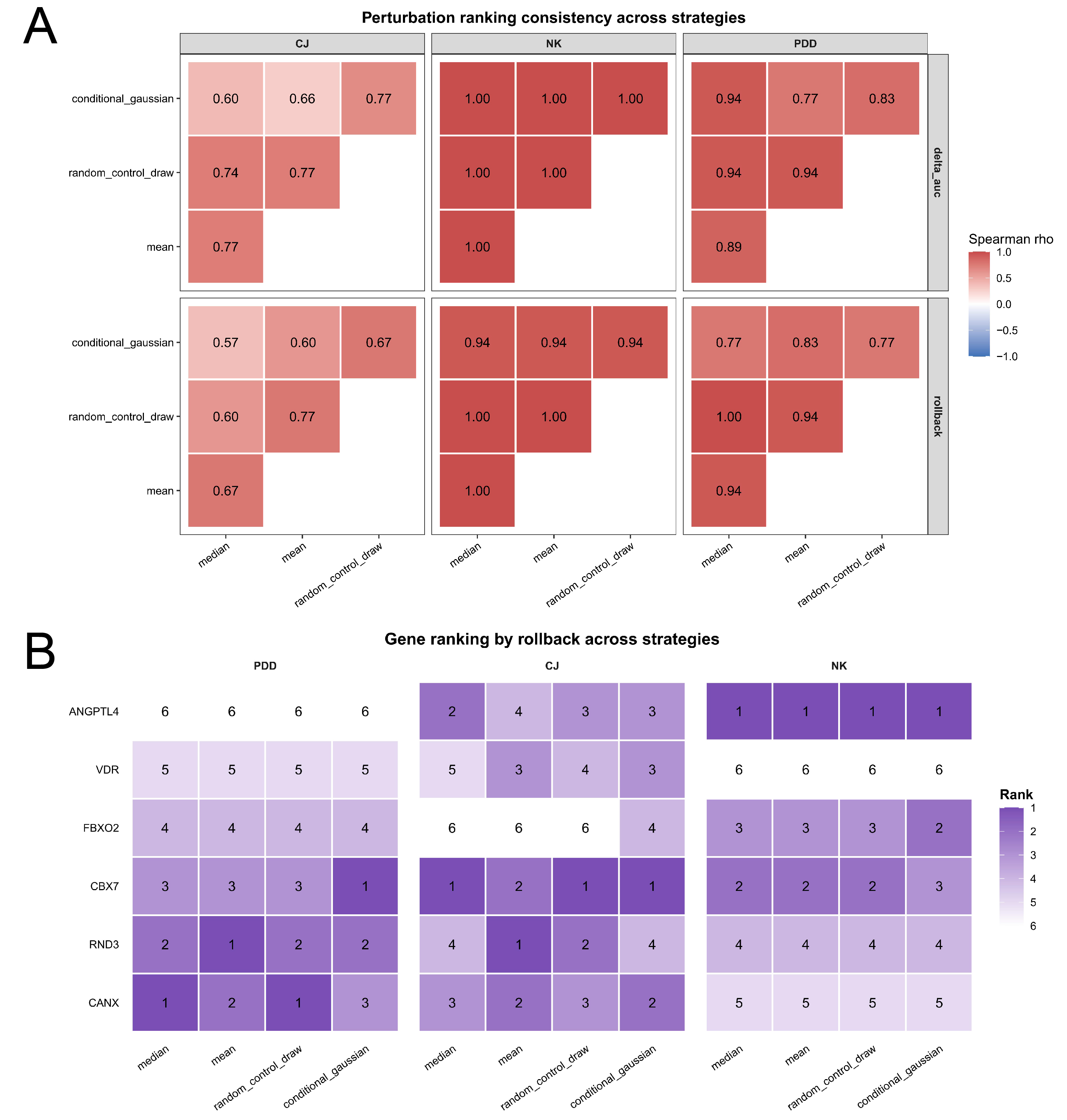

Supplement: Supplementary file 1 [file animals-16-01510-s001.zip › Supplementary Figures/Fig. S5.jpg]

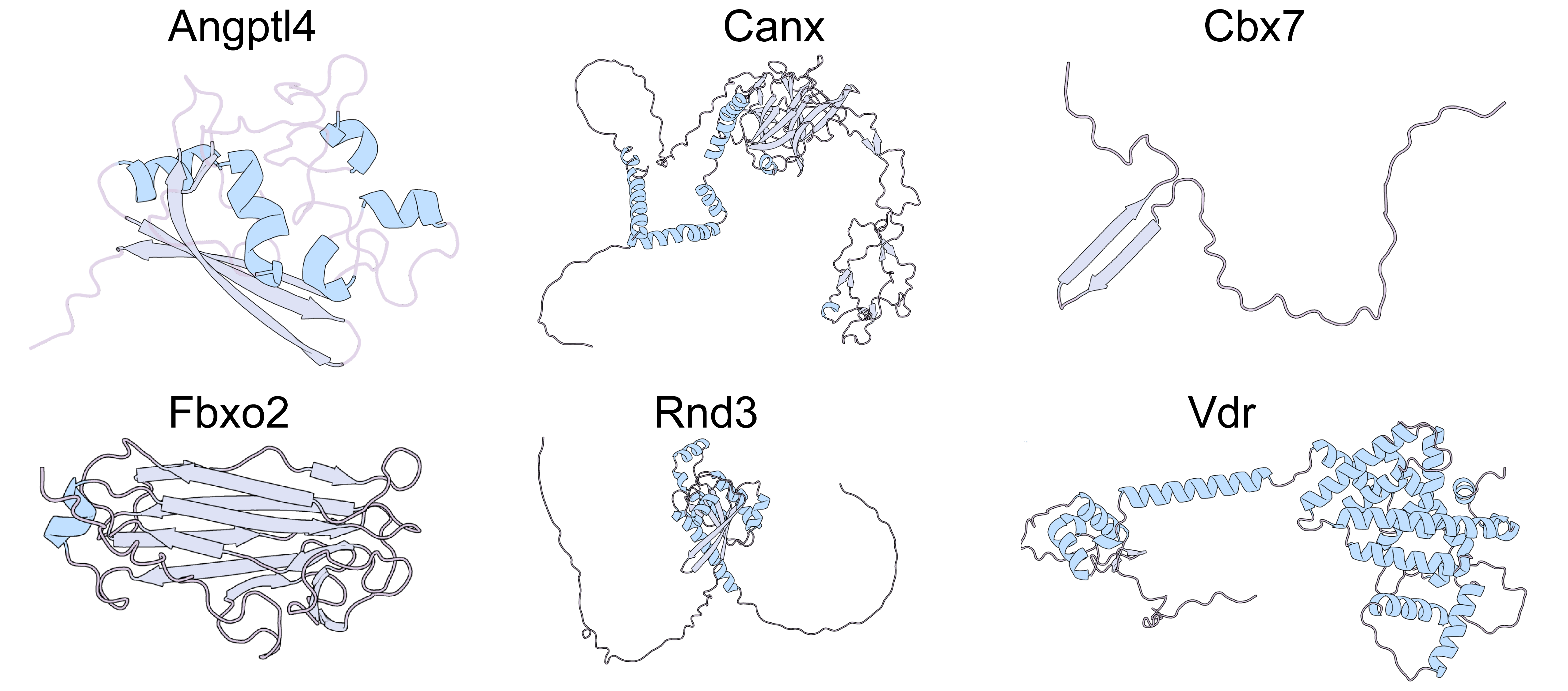

Supplement: Supplementary file 1 [file animals-16-01510-s001.zip › Supplementary Figures/Fig. S6 .tif]

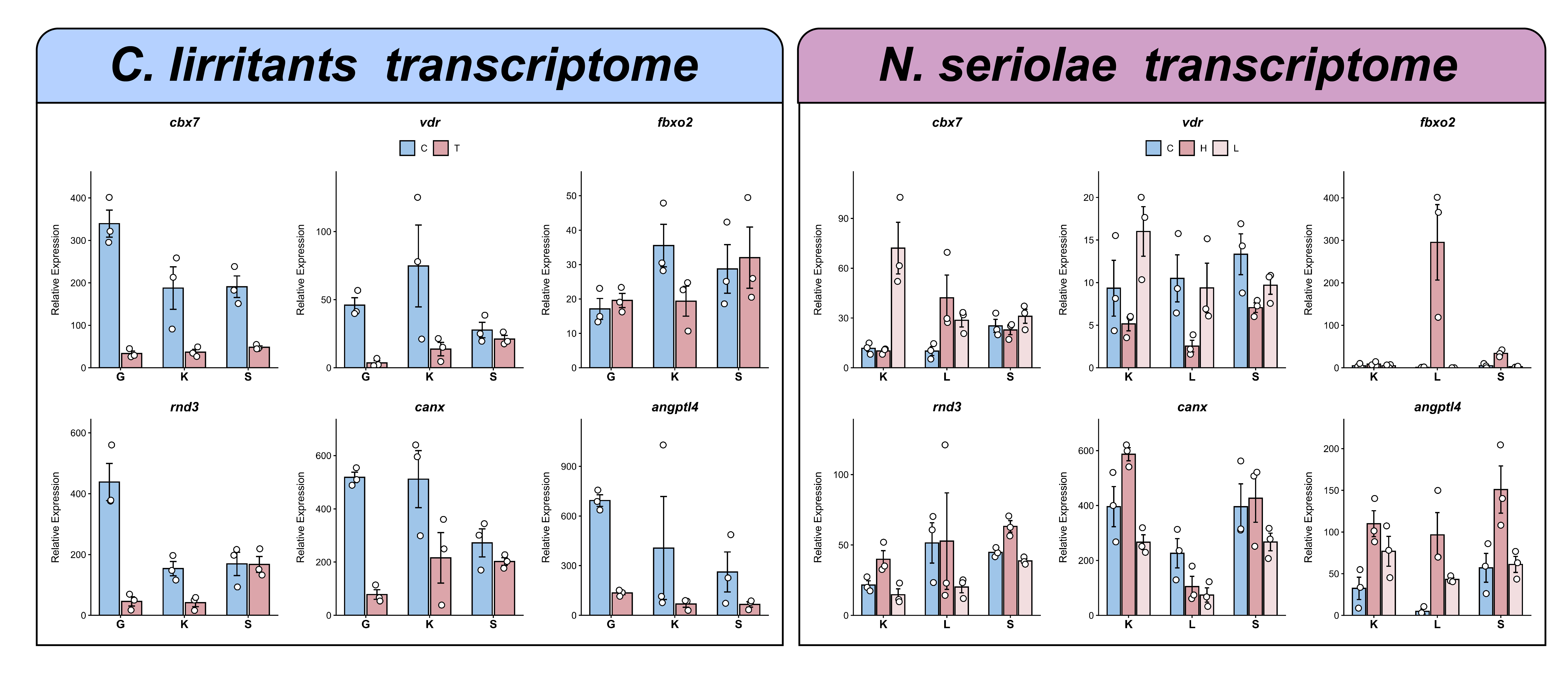

Supplement: Supplementary file 1 [file animals-16-01510-s001.zip › Supplementary Figures/Fig. S7.jpg]

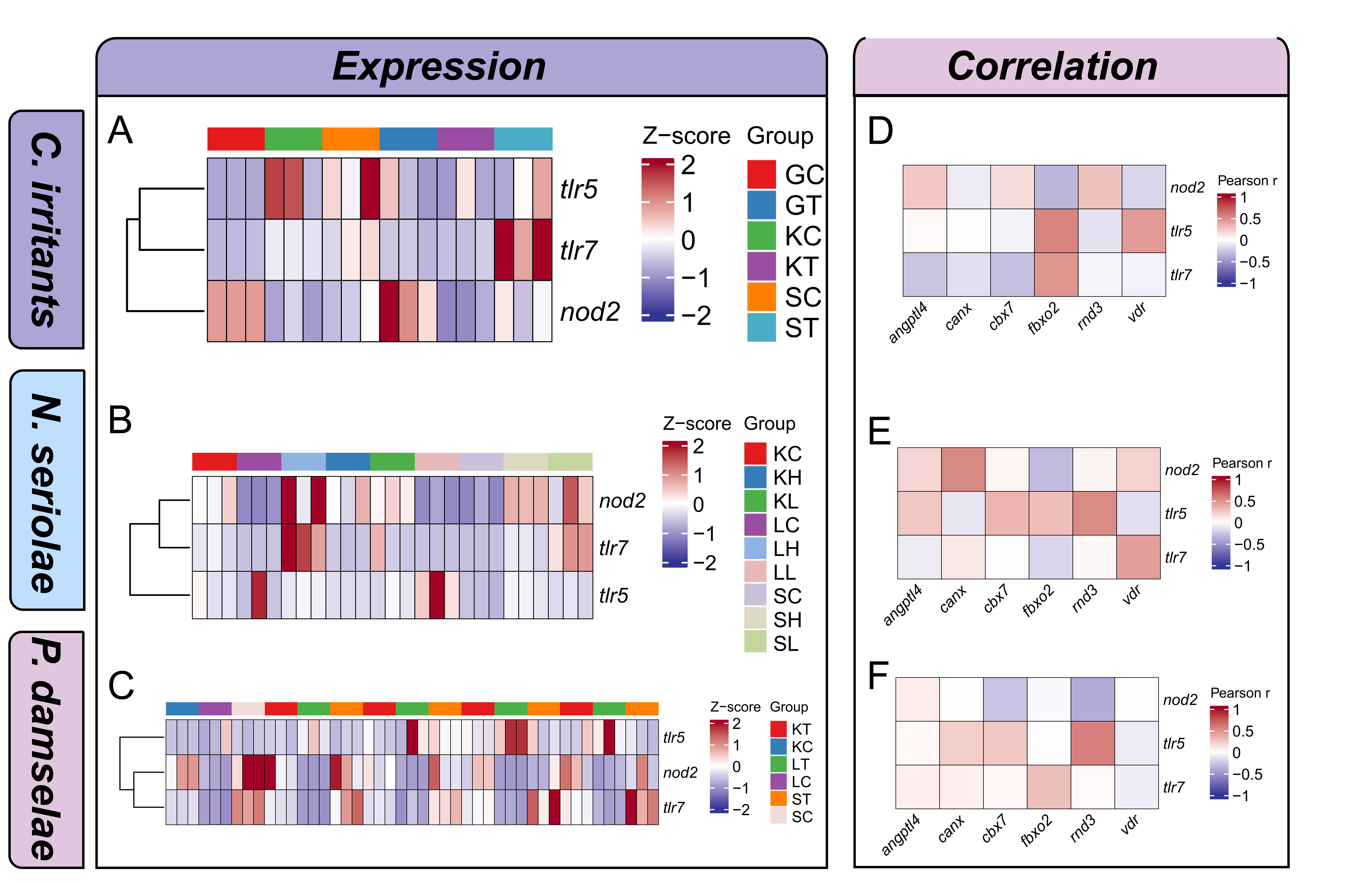

Supplement: Supplementary file 1 [file animals-16-01510-s001.zip › Supplementary Figures/Fig. S8 .tif]
